# Design rules for controlling active topological defects

**DOI:** 10.1073/pnas.2400933121

**Published:** 2024-05-15

**Authors:** Suraj Shankar, Luca V. D. Scharrer, Mark J. Bowick, M. Cristina Marchetti

**Affiliations:** ^a^Department of Physics, Harvard University, Cambridge, MA 02138; ^b^Department of Physics, University of Michigan, Ann Arbor, MI 48109; ^c^Department of Physics, University of California, Santa Barbara, CA 93106; ^d^Department of Physics, The University of Chicago, Chicago, IL 60637; ^e^Kavli Institute for Theoretical Physics, University of California, Santa Barbara, CA 93106

**Keywords:** active matter, control, topological defects, liquid crystals, fluid dynamics

## Abstract

Topological defects play a central role in the physics of many materials, including magnets, superconductors, and liquid crystals. In active fluids, defects become autonomous particles that spontaneously propel from internal active stresses and drive chaotic flows stirring the fluid. The intimate connection between defect textures and active flow suggests that properties of active materials can be engineered by controlling defects, but design principles for their spatiotemporal control remain elusive. Here, we propose a symmetry-based additive strategy for using elementary activity patterns, as active topological tweezers, to create, move, and braid such defects. By combining theory and simulations, we demonstrate how, at the collective level, spatial activity gradients act like electric fields which, when strong enough, induce an inverted topological polarization of defects, akin to a negative susceptibility dielectric. We harness this feature in a dynamic setting to collectively pattern and transport interacting active defects. Our work establishes an additive framework to sculpt flows and manipulate active defects in both space and time, paving the way to design programmable active and living materials for transport, memory, and logic.

## Significance

Active fluids, such as bacterial suspensions and flowing tissues, often exhibit orientational order disrupted by topological defects. Much is understood about how internal driving powers nonequilibrium dynamics, but how can we design protocols to transport and organize patterns in active fluids for functional goals? We develop an additive symmetry-based framework to control the dynamics of such topological defects by spatiotemporally manipulating active stresses. Our framework identifies design principles for controlling defect trajectories using active tweezers, as well as collections of interacting defects using patterns of activity. By combining simulations with theory, we uncover necessary symmetry conditions and trade-offs that govern defect control policies in active media, suggesting general rules for manipulating a broad range of synthetic and biological active matter.

The ability to manipulate matter at the nano-, micro-, and mesoscale is essential for developing functional materials with adaptive and responsive properties (1). A common strategy is to employ repetitive assembly of discrete physical units (e.g., polymers, colloids, elastic beams, etc.) to construct (meta)materials that acquire novel morphologies and functionalities from their architecture (1–5). When rationally designed, such structured materials exhibit unconventional mechanical responses (5), enable programmable computation in materia (6, 7) and mimic living systems (4). But this approach does not apply to systems with mesoscale order that behave as continuous media. An alternate strategy is to use naturally localized excitations, such as topological defects, as discrete building blocks of a hierarchical material.

Topological defects are characteristic singularities that emerge when ordered phases of matter are rapidly quenched or are frustrated by boundaries and external fields (8). These discrete excitations encode information of the continuous order parameter in robustly localized singularities that behave as effective quasiparticles. From manipulating magnetic skyrmions (9, 10) and superconducting vortices (11, 12) to knotting disclination loops in liquid crystals (13–15), the control of defects in diverse systems offers new opportunities for designing reconfigurable memories and logic devices.

**Fig. 1.**
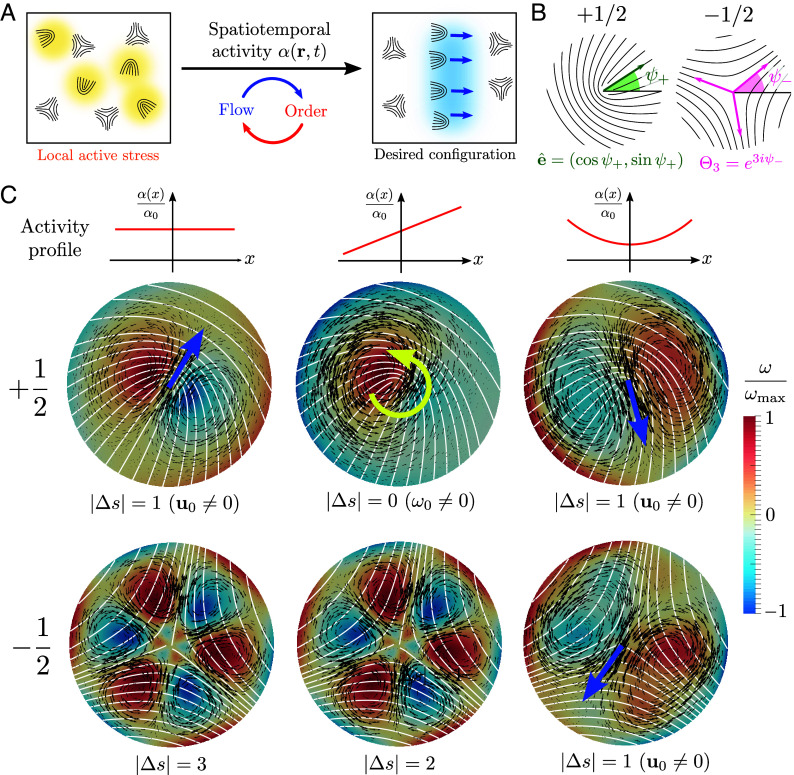
Additive framework for spatiotemporal control of active defects. (*A*) Active stresses generate flow through distortions of order in active nematic fluids resulting in the proliferation of motile defects. How can we locally actuate active stresses to achieve desired defect textures and flow patterns in space-time? (*B*) Disclinations with topological charge ν=±1/2 have distinct symmetries characterized by a vector $\hat{e}$ (+1/2) and a complex triatic parameter $\Theta_{3}$ (−1/2). (*C*) In the vicinity of a defect (director, white lines), simple polynomial activity profiles (constant: *Left*, linear: *Middle*, quadratic: *Right*) in space locally generate distinct flow (u, black arrows) and vorticity ($\omega =\hat{z}\cdot \left(\nabla \times u\right)$, heat map) fields, here computed using Eq. **2** with a screening length $\ell_{\eta }=\sqrt{\eta /\Gamma }=2\xi$ and no-slip boundary conditions. The nature of the flow velocity and vorticity at the defect core ($u_{0}$, blue arrow; $\omega_{0}$, yellow arrow) is dictated by the combined rotational symmetry of the defect texture (s) and the activity pattern (n) quantified by the absolute index difference |Δs|=|s−n|. Linear combinations of individual activity patterns simply sum the respective flow fields providing an additive strategy for generating arbitrarily complex active flows.

Topological defects also naturally emerge in active fluids (16–18), i.e., fluids composed of self-driven units such as bacteria, motor protein-biofilament constructs, active colloids, and living cells (19–23) that can organize into states with (nematic) liquid crystalline order. Elementary defects in two-dimensional (2D) nematics are disclinations characterized by a topological winding number ν=±1/2 (8). In active nematics, spontaneous flows are driven by self-propelled +1/2 defects (18, 24, 25) that chaotically self-stir the fluid (26–28). The intimate feedback between distortions of order, dynamical defects, and flow (Fig. 1*A*) makes active fluids an attractive platform for manipulating transport of matter, energy, and information far from equilibrium (18, 29, 30). Global control of active flows has been achieved by imposing constraints via geometry, confinement, substrates, etc. (31–36). More recent experimental advances in optically responsive platforms now allow local spatiotemporal control of internal stresses in bacterial and synthetic active fluids (37–42) enabling unprecedented abilities to sculpt and configure active materials on demand. Complementing these experimental results, recent theoretical works have also begun exploring the inverse problem in the context of optimal control (43, 44), reinforcement learning (45), and pattern formation (46–49).

But how can we construct spatiotemporal profiles of active stresses to dynamically control active defects and flow (Fig. 1*A*)? Current approaches largely focus on controlling the motion of individual +1/2 defects by using geometric patches of constant activity (41, 47, 50). Optimal control policies generate complex dynamical activity profiles (43) that often lack clear interpretation. Other studies have explored the response of +1/2 defects to linear ramps and step jumps in activity (41, 47, 51, 52), but a systematic quantification of defect patterns as a function of activity gradients is absent (see refs. 46 and 48 for recent efforts). Furthermore, none of the existing approaches allow −1/2 defects to be controlled. A rational design framework to control both ±1/2 topological defects using activity is lacking.

Here we systematically solve this inverse problem by developing a symmetry-based additive approach (Fig. 1) that exploits the linearity of inertia-less dynamics. A key result is the derivation of a selection rule which provides the necessary symmetry conditions that an activity pattern must satisfy to generate any desired ±1/2 motion. The usefulness of these rules is demonstrated in numerical simulations by designing active topological tweezers that can manipulate complex defect trajectories. At the many-body level, a hydrodynamic approach generalizes these results to include defect interactions and helps understand both the static and dynamic response of an interacting defect gas to activity gradients, that we numerically quantify. Altogether, our framework unifies existing results and provides general design principles for controlling arbitrary charge defects in uniaxial active fluids.

## Nematodynamics with Spatiotemporal Activity

We model the active fluid as a thin, two-dimensional (2D) viscous layer with a nematic order parameter Q and flow velocity u whose coupled dynamics is governed by Stokesian force balance and passive relaxation, which give (16–18)

[1] $$\begin{array}{cc} & \partial_{t}Q+u\cdot \nabla Q=S\left(u,Q\right)+\frac{1}{\gamma }H, \end{array}$$

[2] $$\begin{array}{cc} & -\Gamma u+\eta \nabla^{2}u-\nabla \Pi +\nabla \cdot \left(\sigma^{a}+\sigma^{\mathrm{el}}\right)=0. \end{array}$$

Local order is advected, rotated, and sheared by flow (captured by S(u,Q); see *Materials and Methods* for details) and distortions relax via the molecular field $H=\left(a_{2}-a_{4}\;\text{tr}\left[Q^{2}\right]\right)Q+K\nabla^{2}Q$ ($a_{2,4}>0$) with elasticity K and rotational viscosity γ (Eq. **1**). Force balance (Eq. **2**) includes damping from viscosity (η) and friction (Γ), pressure Π enforcing incompressibility (∇·u=0), an elastic stress $\sigma^{\mathrm{el}}$ (see *Materials and Methods* for details) and an active stress $\sigma^{a}=\alpha Q$ (16), where the activity α captures the average strength of the oriented force dipoles exerted by the active units on the fluid (α<0: extensile, α>0: contractile). Importantly, activity α(r,t) varies in space and time, serving as the control variable and we focus on the extensile case (α<0) relevant for most experimental systems (17, 21) (though our results hold more generally). In the following, we work in units such that the nematic correlation length $\xi =\sqrt{K/a_{2}}=1$, the nematic relaxation time $\tau_{n}=\gamma /a_{2}=1$, and a passive elastic stress scale KΓ/γ=1

## Controlling Isolated Active Defects

### Symmetry-Based Selection Rule.

As any distortion of order generates flow in an active fluid (Eq. **2**), defects self-generate local active flows that advect and rotate them (24, 25, 53). By assuming a separation of timescales wherein nematic distortions relax faster than the dynamics of defects (24, 25, 53), we can neglect the nonlinearity in Eq. **1**. Thus to control the trajectory of an individual defect described by 3 independent degrees of freedom (DoFs, two translational, one rotational), we need to prescribe the local flow velocity $u_{0}$ and vorticity $\omega_{0}$ actively generated at the defect core. But the control parameter, the activity α(r,t), is a single scalar field with effectively infinite DoFs. To overcome this DoF mismatch, reminiscent of similar problems in neuromotor control (54), we turn to symmetry.

Both ±1/2 defects are distinguished by the geometry of their local nematic texture; comet-shaped +1/2 defects have a polarity captured by a unit vector $\hat{e}$ (Fig. 1*B*, *Left*), while triangular −1/2 defects have a three-fold symmetry captured by a unit complex triatic parameter $\Theta_{3}$ (Fig. 1*B*, *Right*) (55, 56); see *SI Appendix* for details. In the simple case of constant activity, it is well known that +1/2 defects self-propel along their polarity with nonzero flow at their core ($u_{0}^{+}\propto \alpha \hat{e}$) while three-fold symmetric −1/2 defects do not ($u_{0}^{-}=0$) (17, 18), highlighting the importance of defect rotational symmetry for its motion. For a generic activity profile, because the active stress $\sigma^{a}$ is bilinear in activity (α, the control) and the nematic texture (Q, the state), the combined symmetry of the two fields dictates the nature of local flow. Euclidean isometries of the plane that leave the origin (defect core) fixed are characterized by the orthogonal group O(2) and all its discrete dihedral subgroups $D_{n}$ (i.e., the symmetries of an n-sided regular polygon). We thus expand the activity in terms of angular Fourier harmonics ($\alpha \left(r\right)=\sum_{n}{\tilde{\alpha }}_{n}\left(r\right)e^{in\phi }$, ϕ is the polar angle) which offer a natural basis with definite n-fold dihedral symmetry. The rotational symmetry of 2D nematic defects can be similarly quantified. Defect textures with topological winding ν can be assigned an integer symmetry index s=2|1−ν| (56) corresponding to their dihedral symmetry group $D_{s}$. As expected, +1/2 defects have s=1 and −1/2 defects have s=3.

To obtain a finite defect velocity or vorticity ($u_{0},\omega_{0}\neq 0$), the angle average (zeroth angular moment) of the respective fields must be nonvanishing. Linearity of Eq. **2** and the form of the active stress $\sigma^{a}$ then prescribe a simple selection rule (see Theorem 3.1 in *SI Appendix* for proof): for a defect with symmetry s subjected to an n-fold symmetric activity profile (only ${\tilde{\alpha }}_{\pm n}\neq 0$), a necessary condition for self-propulsion ($u_{0}\neq 0$) is |s−n|=1, and for self-rotation ($\omega_{0}\neq 0$) is |s−n|=0 (Fig. 1*C*). While symmetry dictates the existence (or not) of local active flow, its specific form and direction depend on details of the activity pattern and defect orientation. An explicit calculation of $u_{0}$, $\omega_{0}$ for ±1/2 defects in a smoothly varying activity profile yields (see *SI Appendix* for details)

[3] $$\begin{array}{cc} & u_{0}^{+}=V_{+}\cdot \hat{e},\;\omega_{0}^{+}=\hat{z}\cdot \left(\Omega_{+}\times \hat{e}\right), \end{array}$$

[4] $$\begin{array}{cc} & u_{0}^{-}=\left(\;\text{Re}\left[\Theta_{3}V_{-}\right],\;\;\text{Im}\left[\Theta_{3}V_{-}\right]\right),\;\omega_{0}^{-}=-\;\text{Re}\left[i\Theta_{3}\Omega_{-}\right], \end{array}$$

where (to leading order in gradients) $V_{+}\propto \alpha I+O\left(\nabla^{2}\alpha \right)$, $V_{-}\propto \partial^{2}\alpha$ ($\partial =(\partial_{x}-i\partial_{y})/2$), $\Omega_{+}\propto \nabla \alpha$ and $\Omega_{-}\propto \partial^{3}\alpha$ are translational and rotational response coefficients that depend linearly on the local activity and its symmetry mandated gradients evaluated at the defect core (see *SI Appendix* for details). In Eqs. **3** and **4**, we have simply Taylor-expanded the activity near the defect core upon assuming weak spatial gradients. Flows generated by ±1/2 defects in elementary polynomial profiles of activity are shown in Fig. 1*C*. Strikingly, we see that while linear gradients of activity (∇α≠0; n=1 symmetry) only contribute to vorticity of +1/2 defects, consistent with (48, 51, 52), quadratic activity gradients (∇∇α≠0; n=2 symmetry) generate net active flow for both ±1/2 defects (Fig. 1*C*) as predicted by the symmetry selection rule. Arbitrary linear combinations of activity patterns then simply superpose their respective flow fields thereby providing an attractive additive and modular strategy for designing complex local flows from active defects.

### Active Topological Tweezers.

**Fig. 2.**
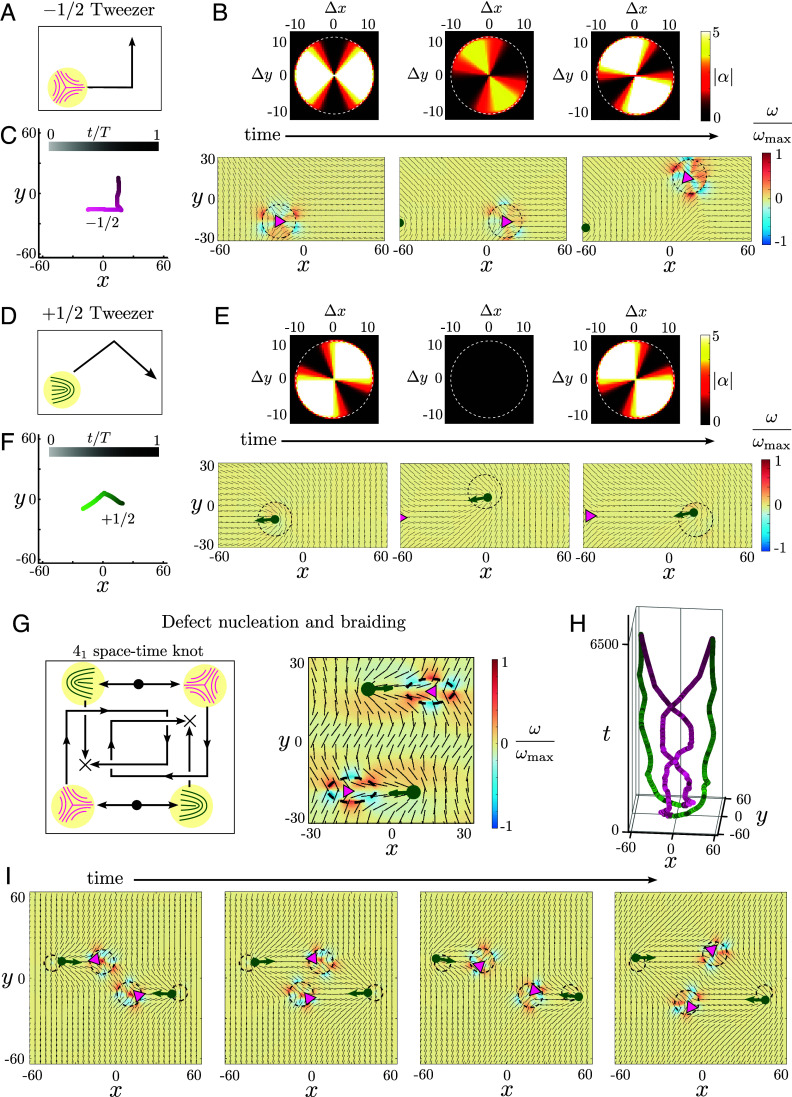
Active topological tweezers enable complex manipulation of defect paths. (*A*–*C*) Moving a −1/2 defect using an active tweezer; see Movie S1 (path shown in *A*). (*B*) The −1/2 defect (magenta triangle) tracks the motion of a small activation disc (dashed circle) with a centered quadrupolar activity profile (see Eq. **5** and *SI Appendix* for details) whose orientation controls the local flow and direction of motion. (Δx,Δy) denote distance from the core of the controlled defect. (*C*) Tracked trajectory of −1/2 defect shaded by the normalized time t/T (T is the protocol duration). (*D*–*F*) Moving a +1/2 defect using an active tweezer; see Movie S2 (path shown in *D*). (*E*) The +1/2 defect (green arrow) tracks the motion of a small activation disc (dashed circle) with a centered quadrupolar activity profile (see Eq. **5** and *SI Appendix* for details). (*C*) Tracked trajectory of +1/2 defect shaded by normalized time t/T. (*G*) Demonstration of simultaneous multidefect control using active tweezers, with defect pair creation (filled circles, *Left*), braided trajectories and pair exchange (arrows, *Left*), and ending in pair annihilation (crosses, *Left*); see Movie S3. An elliptic localized patch of high activity allows local nucleation of ±1/2 defect pairs (right, zoomed-in snapshot). (*H*) Tracked trajectory of defects (+1/2: green, −1/2: magenta) forms a closed braid, the figure-eight ($4_{1}$) knot, in space-time. (*I*) Snapshots showing defect braiding in time.

We now deploy our design framework in numerical simulations of Eqs. **1** and **2** (see *Materials and Methods* and *SI Appendix* for details) to construct activity patterns for basic defect-based operations. For controlling individual defects, we generalize “topological tweezers” (57) used in colloidal crystals to devise active topological tweezers (Fig. 2), i.e., a disc of finite activity, whose motion and local spatiotemporal pattern is chosen to achieve a prescribed defect transport task.

The tweezer design is constrained by the following three conditions: i) the selection rule for the symmetry of the activity pattern, ii) that the maximum activity is smaller than the bend-instability threshold ($\left|\alpha |≲\left(K\Gamma /\gamma \right)\min(1,{\left(\pi \ell_{\eta }/R\right)}^{2}\right)$, where $\ell_{\eta }=\sqrt{\eta /\Gamma }$ is the screening length and R is the tweezer radius), and iii) α does not change sign (so the system never switches from extensile to contractile or vice versa). To reduce the effort of actuation, we minimize gradients in activity, so for simplicity, we choose radially constant activity profiles as far as we can and choose the smallest angular variation required to satisfy the selection rule. These design principles allow the construction of simple topological tweezers for translating ±1/2 defects.

The selection rule dictates that a quadrupolar or twofold symmetric activity profile (${\tilde{\alpha }}_{\pm 2}\neq 0$) is the simplest pattern that can translate ±1/2 defects in different directions (see *SI Appendix* for details). The simplest choice for α (centered on the defect) that satisfies the above constraints is

[5] $$\begin{array}{c} \alpha \left(r\right)=\left\{\begin{array}{c} \alpha_{0}\left[1+A\sin\left(2\phi \right)+B\cos\left(2\phi \right)\right]\;\left(r\leq R\right)\\ 0\;(r>R) \end{array}\right., \end{array}$$

where ϕ is the polar angle about the defect, $\alpha_{0}<0$ is overall (extensile) activity and different choices of A,B (with $\sqrt{A^{2}+B^{2}}\leq 1$) dictate different flow patterns for both ±1/2 defects (see *SI Appendix*, Figs. S1 and S2 for details).

We illustrate this idea by using an active tweezer with a time-varying quadrupolar activity profile to move a −1/2 defect along a bent trajectory (Fig. 2*A*–*C* and Movie S1). A $90^{{}^{\circ}}$ rotation of the profile causes the −1/2 defect to move in an orthogonal direction. A +1/2 defect can also be moved along a similar trajectory using a different active tweezer profile (Fig. 2*D*–*F* and Movie S2). In both cases, the defect successfully tracks the motion of the tweezer (Fig. 2 *C* and *F*) for a range of disc speeds V that are comparable or smaller than the activity induced speed $|u_{0}|$, i.e., $V\leq |u_{0}|$, but very rapid disc motion ($V\gg |u_{0}|$) leaves the defect behind; see *SI Appendix* for details on tweezer protocols (*SI Appendix*, Tables S1–S5) and their characterization (*SI Appendix*, Figs. S3 and S4).

More complex defect manipulations are also possible. As an example, we implement an active tweezer-based protocol for simultaneous multidefect control and use it to accomplish a nontrivial defect exchange and braiding task (Fig. 2*G*–*I* and Movie S3). In a uniformly ordered nematic, we nucleate two pairs of ±1/2 defects using a high activity ramp within a localized elliptic patch that controls the initial orientation of the defect pair created (Fig. 2*G*, *Right*). After the defect pairs are separated, the −1/2 defects are braided around each other using active tweezers and finally annihilated with the +1/2 defect from the opposite pair, accomplishing pair exchange (Fig. 2 *G* and *I* and Movie S3). Although defects also experience elastic forces due to distortions of the nematic (8), the local active forces are sufficiently strong to overcome any elastic interaction. The world lines of the four defects, from creation to annihilation, trace out a closed braid with four crossings forming the figure eight ($4_{1}$) knot in space-time (Fig. 2*H*), which is the simplest, yet nontrivial, achiral prime knot. Notably, unlike active nematics with homogeneous activity, where the spontaneous motility of +1/2 defects alone drives autonomous braiding of defect trajectories (26), our example in Fig. 2*G*–*I* demonstrates braiding of the usually disregarded −1/2 defect.

Active tweezers hence generate patterned flows that enable control of arbitrarily complex braided and knotted trajectories for both ±1/2 defects. These capabilities suggest potential strategies for controlling local fluid mixing (26, 28) and provide key steps toward developing reconfigurable space-time assemblies of active defects for programmable logic devices (50, 58).

## Controlling Interacting Defect Collectives

### Active Defect Hydrodynamics.

Having demonstrated the capability for controlling individual or a few defects, we extend our framework to address multidefect control at the collective level. To do so, we need to account for two additional features. The first is elastic forces between defects that take the form of Coulomb interactions, familiar from passive liquid crystal physics (8). The second is the well-known propensity of active nematics to develop chaotic flows and active turbulence (for sufficiently high activity, |α|≫KΓ/γ), accompanied by swirling ±1/2 defect pairs that spontaneously unbind and proliferate (17, 18). In the regime of many such unbound defects, a coarse-grained hydrodynamic approach for the defect gas is warranted. Following previous work by some of us (25, 46), we develop effective defect hydrodynamic equations that average over fluctuations on the scale of the mean defect spacing and the defect lifetime to describe the distribution of ±1/2 defects in terms of smoothly varying fields such as their respective densities $\rho_{\pm }\left(r,t\right)=\left\langle \sum_{i}\delta \left[r-r_{i}^{\pm }\left(t\right)\right]\right\rangle$ ($r_{i}^{\pm }$ is the position of the ith ±1/2 defect; see *SI Appendix* for details).

A key advance over ref. 46 is the inclusion of both a polarization field $p\left(r,t\right)\;=\;\langle \sum_{i}{\hat{e}}_{i}\delta \left[r-r_{i}^{+}\left(t\right)\right]\rangle$ and a complex triatic order parameter $T_{3}\left(r,t\right)=\left\langle \sum_{i}\Theta_{3}^{i}\delta \left[r-r_{i}^{-}\left(t\right)\right]\right\rangle$ to capture collective orientational ordering of ±1/2 defects due to spatial activity gradients (see *SI Appendix* for details). We derive defect hydrodynamics by coarse-graining effective active particle-like dynamics for ±1/2 defects (see *SI Appendix* for details) that combine activity gradient induced motility and rotations from Eqs. **3** and **4** with passive elastic interactions and active collective torques (similar to previously derived torques in refs. 25 and 59). Defect interaction forces and torques are mediated by nematic distortions quantified by the smoothed phase gradient $v_{n}=\left\langle \nabla \theta \right\rangle$, where θ is the local nematic orientation (see *SI Appendix* for details).

For a static activity profile α(x) varying in 1D, at steady-state we can set $\rho_{\pm }=\rho_{\pm }\left(x\right)$, $p=p\left(x\right)\hat{x}$, $v_{n}=v_{n}\left(x\right)\hat{y}$ etc. (see *SI Appendix* for details). Neglecting any nonlinear buildup of defect order and expanding to lowest order in gradients, the hydrodynamic equations governing collective flux balance for the ±1/2 defect orientations and their velocities then reduce to (see *SI Appendix* for details)

[6] $$\begin{array}{cc} & -\frac{1}{\tau_{R}}p-\frac{1}{2}\partial_{x}\left(\rho_{+}V_{+}\right)-\frac{1}{2}\rho_{+}\Omega_{+}+\mu_{R}V_{+}\rho_{+}\;v_{n}=0, \end{array}$$

[7] $$\begin{array}{cc} & -\frac{1}{\tau_{R}}T_{3}-\frac{1}{2}\partial_{x}\left(\rho_{-}V_{-}\right)+\mu_{R}V_{-}\rho_{-}\;v_{n}=0, \end{array}$$

[8] $$\begin{array}{cc} & \;\;\;pV_{+}-T_{3}V_{-}+2\mu Kn\;v_{n}=0, \end{array}$$

where the 1D response coefficients $V_{\pm }\left(x\right)$, $\Omega_{+}\left(x\right)$ (Eqs. **3** and **4**) are spatial functions of activity (see *SI Appendix* for details), $\tau_{R}$ is a defect reorientation time and $\mu_{R}$ (dimensionless) and μ∝1/γ are rotational and translational defect mobilities respectively (see *SI Appendix* for details). The average defect density $n=(\rho_{+}+\rho_{-})/2$ is controlled by a steady balance of defect creation and annihilation with n(x)∝|α(x)| (60) (see *SI Appendix* for details) and the charge density $\rho =(\rho_{+}-\rho_{-})/2$ is simply obtained from the conservation of topological charge via Gauss’ law: $2\pi \rho \left(x\right)=\partial_{x}v_{n}\left(x\right)$ (46). Note, Eq. **8** balances the motility ($V_{\pm }$) of both ±1/2 defects with elastic forces ($∼\mu Knv_{n}$) to set the topological charge current to zero in steady state. Eqs. **6**–**8** then allow us to compute the spatial distribution of defects in a given 1D activity profile.

### Static Response and Dipole Inversion.

**Fig. 3.**
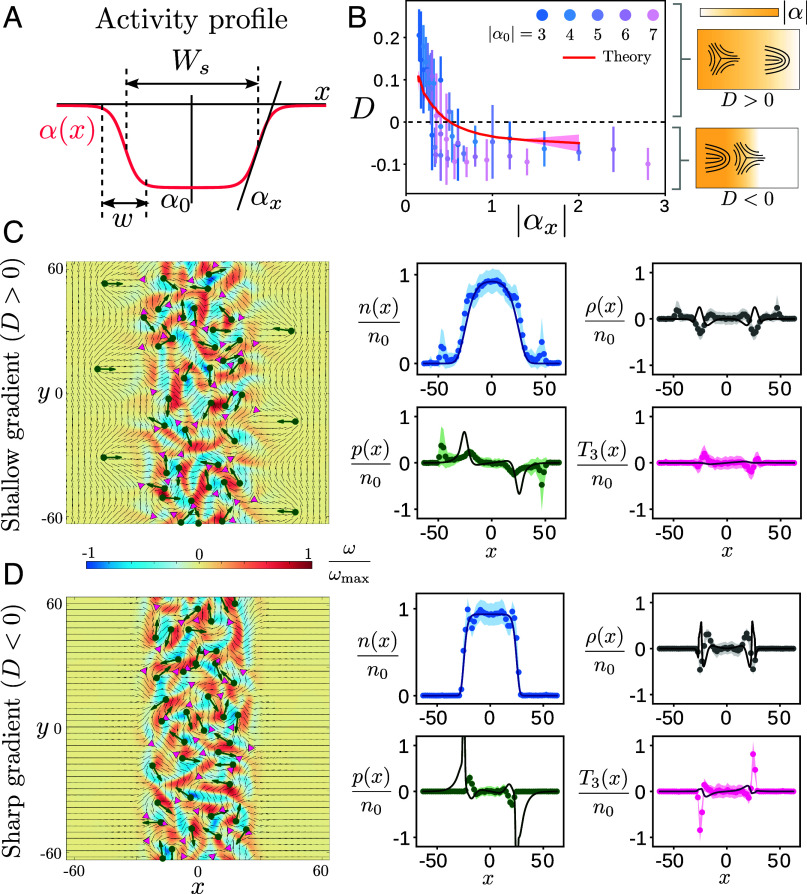
Collective spatial patterning of defects realizes charge dipole inversion. (*A*) A 1D active strip of width $W_{s}=50$ with an extensile activity profile α(x)<0 that smoothly connects the maximal activity $\alpha_{0}$ in the interior of the strip to zero outside. In the interfacial region, the activity varies as a sigmoid over a width w, generating an activity gradient $\alpha_{x}∼\alpha_{0}/w$. (*B*) Topological dipole moment (D) quantifies interfacial charge separation as a function of activity gradient ($\alpha_{x}$) for different $\alpha_{0}$, w (dots: numerical simulation; the error bar is one SD). The model (Eqs. **6**–**8**) quantitatively predicts the dipole flip transition (red line using $|\alpha_{0}|=5$, shaded region is one SD using an N=10 bootstrap subsample; see *SI Appendix* for details). (*C* and *D*) The dynamical steady state for shallow (w=40, *C*) and sharp (w=15, *D*) activity gradients (both with $|\alpha_{0}|=5$) with snapshots shown (*Left*) and spatial defect distributions quantified (*Right*) comparing simulations (dots, shaded region is one SD) with theory (lines; see *SI Appendix* for details). Statistical variation in fitting of model parameters is described in *SI Appendix* and Figs. S5 and S8.

To validate and test our theoretical predictions, we perform numerical simulations of the full nematodynamic model (Eqs. **1** and **2**) using different activity patterns. For simple activity profiles such as a linear or quadratic gradient, we can analytically solve Eqs. **6**–**8** and obtain quantitative fits for n(x), ρ(x), p(x), $T_{3}\left(x\right)$ (see *SI Appendix* and Figs. S5–S8 for details). With the phenomenological parameters of our defect hydrodynamic model (Eqs. **6**–**8**) in hand, we now challenge our model using a more complex activity profile consisting of an active strip of width $W_{s}=50$ flanked by passive regions (Fig. 3*A*). Within the active strip, the maximal activity ($\alpha_{0}$) is always chosen to be well in the regime of active turbulence. Near the interface, the activity varies in a sigmoidal fashion allowing us to tune the interfacial activity gradient ($\alpha_{x}{\equiv \;\text{d}\alpha /\;\text{d}x|}_{W_{s}/2}∼\alpha_{0}/w$) and the width of the interface (w) independently (Fig. 3*A*).

For shallow gradients ($|\alpha_{x}|\leq 0.2$), the motile +1/2 defects escape and accumulate out of the strip, leaving behind −1/2 defects on the inside (Fig. 3*C*, *Left* and Movie S4). As a result, the interface develops a topological charge dipole as the activity gradient behaves as an “electric field” separating defects by topological charge. This effect, first predicted in ref. 46, simply relies on the fact that +1/2 defects behave like active particles, which accumulate where they move slowly, and disregards the negligible propulsion of −1/2 defects in weak gradients ($V_{-}\ll V_{+}$; see *SI Appendix* for details). Remarkably, for sharp activity gradients ($|\alpha_{x}|\geq 0.2$), we find a different behavior, wherein the +1/2 defects are no longer able to tunnel through the interface, and −1/2 defects instead accumulate near the edge of the strip (Fig. 3*D*, *Left* an Movie S5). Similar effects have been noted previously for individual defects (41, 47), but not at the collective level.

To quantify the charge separation of defects, we compute a steady state dipole moment D=(1/2)∫dx|x|ρ(x) and plot it as a function of the interfacial activity gradient $|\alpha_{x}|$ (Fig. 3*B*). Upon varying both the maximal activity ($|\alpha_{0}|$) and the interfacial width (w), we find a common trend, i.e., when the activity gradient $|\alpha_{x}|$ is increased, the dipole moment switches from D>0 (excess +1/2 defects on the low activity side) to D<0 (excess +1/2 defects on the high activity side); see Fig. 3*B*. Note that, as $|\alpha_{0}|\gg 1$ (above the active turbulence threshold), the dipole moment remains nonvanishing even for arbitrarily weak gradients ($|\alpha_{x}|\to 0$, w→∞). Following the electrostatic analogy, the active nematic behaves as an unusual polarizable medium with a nonlinear response such that for shallow activity gradients (“weak field”) the system behaves as a conventional dielectric, whereas for sharp activity gradients (“strong field”), the system displays a negative static susceptibility, a feature forbidden at equilibrium (61).

How can we understand the inversion of the dipole moment? While previous simpler treatments are unable to predict this phenomenon (46), by accounting for the active propulsion and rotation of both ±1/2 defects, our improved defect hydrodynamic model (Eqs. **6**–**8**) quantitatively captures this dipole flip transition without any fitting parameters (Fig. 3*B*, red line using $|\alpha_{0}|=5$; see *SI Appendix* for details). We next compare the numerically measured spatial distributions of defect density (n), charge density (ρ), and orientational order parameters (p, $T_{3}$) with our model predictions, with qualitatively comparable results overall (Fig. 3 *C* and *D*, *Right*). The average defect density n(x) is well predicted in both cases ($n_{0}$ is the maximal defect density in the center) and features of charge separation (ρ(x)) and +1/2 polarization (p(x)) are qualitatively captured for shallow gradients (Fig. 3*C*, *Right* and see *SI Appendix* for details). But the model predictions for spatial profiles are less accurate for sharp gradients (Fig. 3*D*, *Right*), where p(x) is overestimated and $T_{3}\left(x\right)$ is underestimated (see *SI Appendix* for details). We attribute the lack of quantitative accuracy in the spatial profile predictions to the neglect of higher gradient and nonlinear terms that affect the defect density and cause saturation of defect order, particularly for strong activity gradients (see *SI Appendix* for details).

Nonetheless, some general features are apparent. Intuitively, the sharp activity interface acts like a virtual wall that blocks the motion of defects through it, as noted previously in simulations (47) and experiments (36, 41). In a thin boundary layer near the edge of the active strip, the locally strong activity gradient causes −1/2 defects to self-propel faster than +1/2 defects ($V_{-}\geq V_{+}$), but both ±1/2 defects rapidly lose their motility upon crossing the interface. Similar boundary layers have been recently observed at confining walls as well (62). As a result, −1/2 defects get preferentially trapped along the interface, preventing any +1/2 defects from crossing the interface, thus realizing the inverted dipole state.

The resulting charge dipole is accompanied by a interfacial defect ordering and a change in the nematic orientation outside the active strip as well. As shown in Fig. 3 *C* and *D*, while shallow activity gradients cause the nematic to orient parallel to the interface (due to the escaped +1/2 defects whose polarization points down activity gradients), a sharp interface forces the nematic to reorient perpendicular to the interface (due to ordered −1/2 defects). Our results demonstrate that defect-decorated activity interfaces display tunable “active anchoring” (63) that allows control of collective defect organization and nematic orientation simply via the strength of the local activity gradient.

### Dynamic Response and Rectification.

**Fig. 4.**
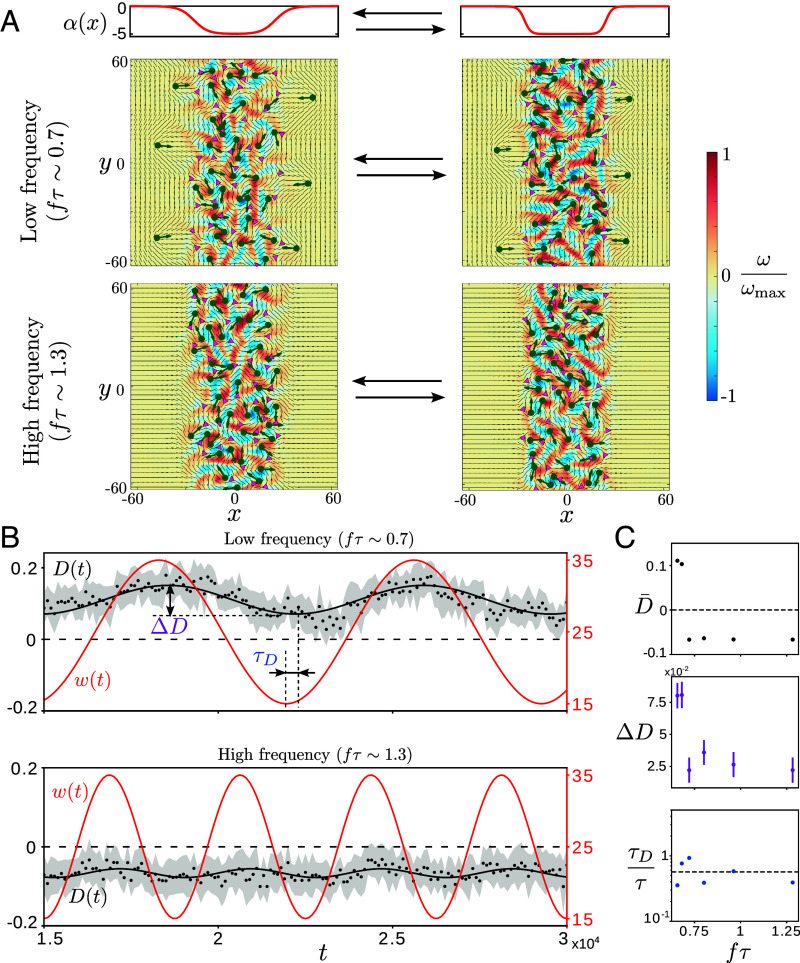
Dynamic response of active defects to oscillatory gradients. (*A*) The interfacial width (w) is varied sinusoidally and its oscillation frequency (f) controls defect organization. (*B*) The dynamical steady state is quantified by a periodic dipole moment D(t) (dots: simulation with shaded region as one SD, black line: sinusoidal fit) which switches from overall D(t)>0 at low frequencies (fτ∼0.7, *Top*) to D(t)<0 at higher frequencies (fτ∼1.3, *Bottom*). The time trace of the imposed interfacial width (w(t)) is shown in red. (*C*) The sinusoidal fit (with frequency f) of the time-varying dipole moment shows how the average value ($\overset{¯}{D}$, *Top*), amplitude of oscillation (ΔD, *Middle*), and time delay ($\tau_{D}$, *Bottom*) vary as a function of drive frequency (f). Error bars from the sinusoidal fit correspond to one SD and are smaller than the marker size when not visible.

We now employ the active strip as a defect patterning motif in a dynamic setting. As shallow interfaces are leaky to the escape of +1/2 defects, but sharp interfaces are not, we ask whether periodically oscillating the interface width allows us to dynamically control the organization of active defects. We fix $|\alpha_{0}|=5$ and vary the interface width sinusoidally in time (with frequency f) between values $w_{\text{min}}=15$ and $w_{\;\text{max}}=35$ (Fig. 4*A*), so that the average width ($\overset{¯}{w}=25$) corresponds to an almost vanishing dipole moment (D≈0) in the static limit (Fig. 3*B*). The average time $\tau ={\overset{¯}{w}}^{2}/D_{a}$ it takes +1/2 defects to cross the interface with an active diffusion constant $D_{a}=\left\langle \right|u_{0}{|}^{2}\rangle \tau_{R}/2∼0.82$ (see *SI Appendix* for details) sets a characteristic time scale that controls the dynamic response. At low oscillation frequencies (fτ∼0.7, with τ∼765), +1/2 defects have sufficient time to escape through the shallow interface and remain trapped in the passive region when the interface becomes sharp (Fig. 4*A* and Movie S6). As a result, the system develops a steady-state dipole moment that is always positive (D(t)>0) and oscillates in sync with the interface (Fig. 4*B*, *Top*), albeit with a time delay ($\tau_{D}$, Fig. 4 *B* and *C*).

For a higher driving frequency (fτ∼1.3), +1/2 defects have insufficient time to escape and they get dynamically trapped to the active strip (Fig. 4*A* and Movie S7). The system then dynamically realizes an inverted dipole state with D(t)<0 (Fig. 4*B*, *Bottom*). We quantify this dynamic transition by performing a sinusoidal fit of the steady-state dipole moment and plot the time-averaged dipole moment ($\overset{¯}{D}$), the amplitude of oscillations (ΔD), and the time delay in the response ($\tau_{D}$) as a function of the driving frequency f (Fig. 4*C*). While $\overset{¯}{D}$ and ΔD show stronger variation upon increasing frequency (with $\overset{¯}{D}$ switching sign), the average time delay $\tau_{D}∼0.6\tau$ is intrinsic to the active defect gas and does not vary systematically with the drive (Fig. 4*C*).

### Collective Transport and Optimal Surfing.

**Fig. 5.**
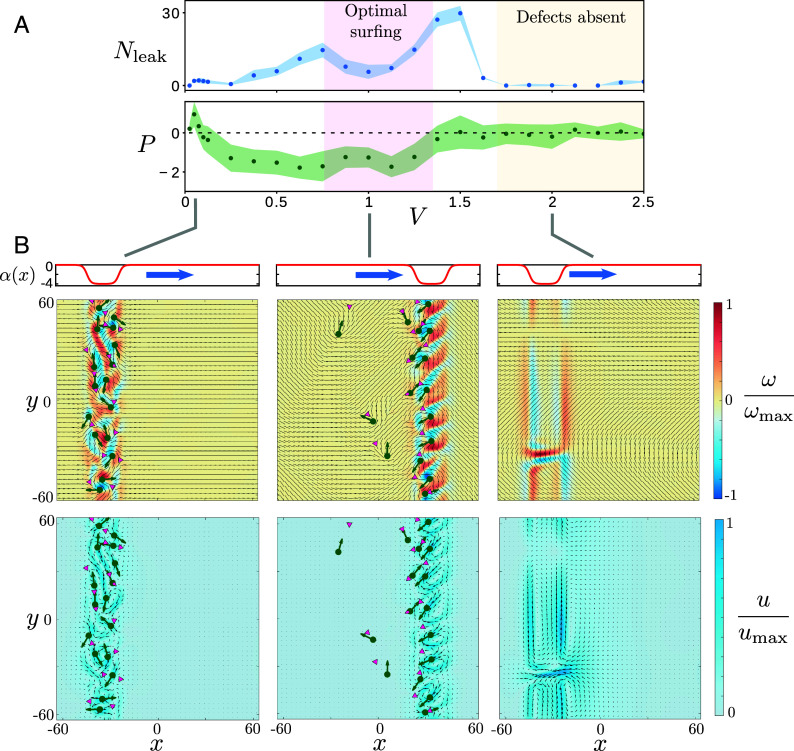
Collective transport of active defects by “surfing.” (*A*) A 1D active strip ($|\alpha_{0}|=4$, $W_{s}=20$, w=10) moving with speed V can be used to collectively transport active defects. Transport efficacy is quantified by the total number of defects that leak out of the strip ($N_{\;\text{leak}}$, blue dots) and the total polarization of +1/2 defects (P, green dots). The blue and green shaded regions represent one SD. Few defects are lost for V≪1 but the transport is slow, whereas for very high speeds (V≫1, yellow shaded region), the strip moves faster than defects can nucleate. Only at intermediate speeds (V∼1, shaded red region) do we obtain optimal defect surfing, where few defects are left behind and the +1/2 defects develop significant collective polarization (|P|≫1). (*B*) Snapshots show the three characteristic regimes for V≪1 (*Left*), V∼1 (*Middle*), and V≫1 (*Right*) with both vorticity (ω) and flow speed (u=|u|) plotted.

Along with defect patterning, another basic task is defect transport. As a simple example, we use a 1D moving active strip (with speed V) to illustrate how active defects can be collectively transported in space. For simplicity, we fix the maximal activity $|\alpha_{0}|=4$ and choose the strip geometry to have sharp interfaces ($W_{s}=20$, w=10) to ensure defects are trapped to the active region when the strip is stationary. To achieve rapid and efficient transport, it is natural to consider large V, but fast motion of the active strip can cause defects to be left behind, despite the sharp interfaces. To quantify this trade-off we compute (in steady-state) the total number of defects that escape and leak out of the active strip (*N*_leak_, Fig. 5*A*) and the net horizontal polarization of all the +1/2 defects (P=∫drp, Fig. 5*A*).

For slow speeds (V≪1) the transport time is long but the defects remain largely localized to the active region ($N_{\;\text{leak}}∼0$) with no net polarization (P∼0) as they follow the traveling strip adiabatically (Fig. 5*B*, *Left* and Movie S8). For very rapid motion of the strip (V≫1, shaded yellow region in Fig. 5*A*), there is insufficient time to nucleate and carry defects (Fig. 5*B*, *Right* and Movie S10). Only for intermediate speeds (V∼1, shaded red region in Fig. 5*A*) do we obtain optimal collective transport of defects characterized by an enhanced carrying capacity (decrease in $N_{\;\text{leak}}$, Fig. 5*A*) and significant +1/2 polarization (|P|≫1, Fig. 5*A*). In this regime, the active strip moves at a speed comparable to the activity-induced propulsion of the defects, allowing the defects to collectively “surf” the imposed traveling wave of activity, and organize the otherwise turbulent interior of the active strip into a state with spatially patterned flow and vortices (Fig. 5*B*, *Middle* and Movie S9).

## Discussion and Conclusion

In this work, we have demonstrated how topological defects offer robust particle-like excitations that can be functionally controlled to manipulate continuous active fluids in space and time. Our symmetry-based additive framework enables the construction of active topological tweezers for basic defect operations that provide the basis of complex computation and logic in a fluidic system (50, 58). By extending the framework to incorporate defect interactions, we developed a coarse-grained hydrodynamic description of active defects at the collective level, enabling the characterization of large-scale patterning and dynamics of the defect gas. Activity gradients are shown to behave as “electric fields” that segregate defects by charge and topologically polarize the active fluid, albeit with unusual responses that mimic certain dielectric metamaterials. Complementing the spatial response, we also probe the dynamic response of defects and highlight simple strategies for patterning and transporting large collections of active defects.

Our work provides a general framework to control and rationally design active materials as metafluids for soft microrobotics (64, 65) and defect-based soft logic (50, 58). Current experiments on light-controlled active fluids (40–42) offer a natural platform to deploy our control strategies. Beyond engineered systems, active defects have been identified in biological tissues and cellular monolayers, and proposed to act as sites of biological function and morphogenesis (66–69). Our work suggests that a similar symmetry-based approach can be used to understand how active defects get functionalized in biological systems, paving the way for controlling and designing defect based autonomous materials from living matter with adaptive and programmable functionality.

## Materials and Methods

Extended data on the details of the numerical modeling and theoretical calculations. Further details on model fitting and analysis are provided in *SI Appendix*.

### Active Nematodynamics.

The orientational order of the active nematic in 2D is locally characterized by an alignment tensor $Q_{\mathrm{ij}}=S\left({\hat{n}}_{i}{\hat{n}}_{j}-\delta_{\mathrm{ij}}/2\right)$ with the director $\hat{n}=\left(\cos\theta ,\sin\theta \right)$, where S is the scalar order parameter and θ refers to the director angle. A continuum hydrodynamic description of the 2D active nematic film is given by (Eqs. **1** and **2**)

[9] $$\begin{array}{cc} & \partial_{t}Q+u\cdot \nabla Q=S\left(u,Q\right)+\frac{1}{\gamma }H, \end{array}$$

[10] $$\begin{array}{cc} & -\Gamma u+\eta \nabla^{2}u-\nabla \Pi +\nabla \cdot \left(\sigma^{a}+\sigma^{\mathrm{el}}\right)=0, \end{array}$$

with the molecular field $H=\left(a_{2}-a_{4}\;\text{tr}\left[Q^{2}\right]\right)Q+K\nabla^{2}Q$ ($a_{2,4}>0$). Note here we have already assumed the system prefers an ordered nematic phase in the absence of activity. Without loss of generality, we set $a_{4}=a_{2}$. Then the equilibrium ground state is an ordered homogeneous nematic with $S=S_{0}=2$. The flow coupling S(u,Q) is given by (70)

[11] $$\begin{array}{cc} S_{\mathrm{ij}} & =Q_{\mathrm{ik}}W_{\mathrm{kj}}-W_{\mathrm{ik}}Q_{\mathrm{kj}}-2\lambda Q_{k\ell }E_{k\ell }Q_{\mathrm{ij}}\\ & \;+\lambda E_{\mathrm{ij}}+\lambda \left(E_{\mathrm{ik}}Q_{\mathrm{kj}}+Q_{\mathrm{ik}}E_{\mathrm{kj}}-\delta_{\mathrm{ij}}Q_{k\ell }E_{k\ell }\right), \end{array}$$

where λ is the flow-alignment parameter (|λ|>1 for flow-aligning systems), $E_{\mathrm{ij}}=\left(\partial_{i}u_{j}+\partial_{j}u_{i}\right)/2$ is the strain-rate tensor (purely deviatoric as ∇·u=0), and $W_{\mathrm{ij}}=\left(\partial_{i}u_{j}-\partial_{j}u_{i}\right)/2$ is the vorticity tensor.

The active stress is $\sigma^{a}=\alpha Q$ (16) and the passive liquid-crystal stress is given by

[12] $$\begin{array}{cc} \sigma_{\mathrm{ij}}^{\mathrm{el}} & =Q_{\mathrm{ik}}H_{\mathrm{kj}}-H_{\mathrm{ik}}Q_{\mathrm{kj}}-K\partial_{i}Q_{\mathrm{kl}}\partial_{j}Q_{\mathrm{kl}}+\lambda \left(2Q_{\mathrm{ij}}+\delta_{\mathrm{ij}}\right)Q_{\mathrm{kl}}H_{\mathrm{kl}}\\ & \;-\lambda H_{\mathrm{ik}}\left(Q_{\mathrm{kj}}+\frac{1}{2}\delta_{\mathrm{kj}}\right)-\lambda \left(Q_{\mathrm{ik}}+\frac{1}{2}\delta_{\mathrm{ik}}\right)H_{\mathrm{kj}}\;. \end{array}$$

The pressure Π enforces incompressibility (∇·u=0) and is calculated from the pressure Poisson equation obtained by taking the divergence of Stokes’ equation (Eq. **10**):

[13] $$\begin{array}{c} \nabla^{2}\Pi =\partial_{i}\partial_{j}\left(\sigma_{\mathrm{ij}}^{\mathrm{el}}+\sigma_{\mathrm{ij}}^{a}\right) \end{array}$$

We note that only symmetric components of $\sigma^{\mathrm{el}}$ will contribute to the pressure, due to the symmetry of the $\partial_{i}\partial_{j}$ operator.

### Numerical Simulations.

In order to numerically simulate the nematodynamic equations, we nondimensionalize Eqs. **9** and **10**. As mentioned in the main text, we employ the nematic coherence length $\xi =\sqrt{K/a_{2}}$ as our unit of length, the elastic relaxation time $\tau_{n}=\gamma /a_{2}$ as our unit of time, and a passive stress scale KΓ/γ as our unit of stress. We are then left with four dimensionless parameters: ${\tilde{\ell }}_{\eta }=\ell_{\eta }/\xi$, $\tilde{\gamma }=\gamma /\eta$, $\tilde{\alpha }=\alpha \gamma /\left(K\Gamma \right)$ and λ. The topological tweezer demonstrations were performed with ${\tilde{\ell }}_{\eta }=5$, chosen to display flow patterns more clearly, while the collective effect simulations were performed at a lower viscosity $\tilde{\ell_{\eta }}=\sqrt{5}$, to increase defect density and improve the statistics of defect averages. For all simulations, we set $\tilde{\gamma }=0.1$ and λ=1.8.

Numerical simulations of the continuum nematodynamic equations (Eqs. **9** and **10**) were performed with a custom Matlab code using a second-order pseudospectral time-exponentiation scheme for numerical integration, on a lattice of 256×256 gridpoints with periodic boundary conditions. We used a grid spacing of dx=0.5 and timestep dt=0.1. The −1/2 and +1/2 tweezer demonstrations in Fig. 2 were run for a total of 1,520 and 1,800 timesteps respectively, while the braiding procedure was run for 6,500 timesteps. The dipole moment datapoints reported in Fig. 3 were computed using N=40 independent simulations, each running for $10^{5}$ timesteps. The data for oscillating activity gradients (Fig. 4) were computed using N=6 independent simulations, each run for $3\times 10^{5}$ timesteps, and the demonstrations with traveling activity patterns (Fig. 5) were run for $5\times 10^{4}$ timesteps. All simulations were performed on an NVIDIA GeForce GTX 1060 Mobile graphics card, and we estimate that each $10^{4}$ timesteps of simulation time corresponded to a wall-clock runtime of ∼12 min, resulting in a typical overall runtime of ∼150 h.

## Supplementary Material

Appendix 01 (PDF)

Movie S1. Demonstration of an active topological tweezer transporting a −1/2 defect (magenta triangle) along a bent trajectory (Fig. 2A-C). Both normalized vorticity (*ω*, top left) and flow speed (*u*, bottom left) are plotted. The activity pattern actuated in the tweezer is shown on the top-right and the defect trajectory is plotted on the bottom-right. The tweezer protocol details are given in Table S1.

Movie S2. Demonstration an active topological tweezer transporting a +1/2 defect (green arrow) along a bent trajectory (Fig. 2D-F). Both normalized vorticity (*ω*, top left) and flow speed (*u*, bottom left) are plotted. The activity pattern actuated in the tweezer is shown on the top-right and the defect trajectory is plotted on the bottom-right. The tweezer protocol details are given in Table S2.

Movie S3. Demonstration of controlled defect pair nucleation, braiding and pair exchange using active topological tweezers (Fig. 2G-I). The normalized vorticity (*ω*), flow speed (*u*) and defect space-time trajectories are plotted. The tweezer protocol details are given in Tables S3-S5.

Movie S4. Collective patterning of defects using an active strip (Fig. 3C) showing both the normalized vorticity (*ω*) and flow speed (*u*), along with the static activity profile (α(*x*)). Shallow interfacial gradients (*w* = 40, *α*_0_ = −5) allow +1/2 defects to escape through and get trapped on the passive sides. This results in a charge polarized interface with a positive dipole moment.

Movie S5. Collective patterning of defects using an active strip (Fig. 3D) showing both the normalized vorticity (*ω*) and flow speed (*u*), along with the static activity profile (*α*(*x*)). Sharp interfacial gradients (*w* = 15, *α*_0_ = −5) entrap −1/2 defects at the interface preventing the escape of motile +1/2 defects. This results in an inverted polarized interface with a negative dipole moment.

Movie S6. Slow dynamic response of defects in an active strip with oscillating gradients (Fig. 4). Both normalized vorticity (*ω*) and flow speed (*u*) are shown along with the dynamic activity profile (*α*(*x, t*)). Slow sinusoidal oscillations of the interface width (fτ ~ 0.7) allow the defects to reorganize quasi-adiabatically and provide enough time for the +1/2 defects to escape outside the strip. This results in a conventionally polarized interface with a time-averaged positive dipole moment.

Movie S7. Rapid dynamic response of defects in an active strip with oscillating gradients (Fig. 4). Both normalized vorticity (*ω*) and flow speed (*u*) are shown along with the dynamic activity profile (*α*(*x, t*)). Fast sinusoidal oscillations of the interface width (f*τ* ~ 1.3) do not provide enough time for the +1/2 defects to escape outside the strip. This results in the defects getting dynamically trapped within the strip leading to an inverted polarized interface with a time-averaged negative dipole moment.

Movie S8. Collective transport of active defects (Fig. 5) with a slowly moving active strip (*V* = 0.025). Both normalized vorticity (*ω*) and flow speed (*u*) are shown along with the dynamic activity profile (*α*(*x, t*)). Slow motion of the strip allows the defect distribution to equilibriate within the strip and get transported quasi-adiabatically. As a result, negligible number of defects leak and escape out of the strip, but the transport task takes a very long time (asymptotically infinite as V → 0).

Movie S9. Optimal collective transport of active defects (Fig. 5) with an active strip moving at intermediate speeds (*V* = 1). Both normalized vorticity (*ω*) and flow speed (*u*) are shown along with the dynamic activity profile (*α*(*x, t*)). At the optimal speed, the motion of the active strip matches the self-propulsion speed of the +1/2 defects allowing the defects to collectively ‘surf’ the travelling activity pattern. This allows the transport task to be completed in a finite time, with minimal leakage and escape of defects from the active strip.

Movie S10. Failed collective transport of active defects (Fig. 5) with a fast moving active strip (*V* = 2). Both normalized vorticity (*ω*) and flow speed (*u*) are shown along with the dynamic activity profile (*α*(*x, t*)). At very large speeds, the activity patterns moves too quickly to provide suffiecient time to even nucleate enough defect pairs and transport them. Any transiently created defect pairs rapidly leave the active region and passively annihilate before they can be transported.

## Data Availability

Code data have been deposited in GitHub (71). All other data are included in the manuscript and/or supporting information.
