# Supplementary material for: Design rules for controlling active topological defects
Source: Proc Natl Acad Sci U S A. 2024 May 15;121(21):e2400933121. doi: 10.1073/pnas.2400933121 (PMC11127047; doi:10.1073/pnas.2400933121)
Supplement: Supplementary file 1 — Appendix 01 (PDF) [file pnas.2400933121.sapp.pdf]

PNAS
www.pnas.org

Supplementary Information for

Design rules for controlling active topological defects

Suraj Shankar, Luca V. D. Scharrer, Mark J. Bowick, M. Cristina Marchetti

Correspondence and requests for materials should be addressed to M. Cristina Marchetti Email: cmarchetti@ucsb.edu and Suraj Shankar Email: surajsh@umich.edu .

This PDF file includes:

Supplementary text
Figs. S1 to S9
Tables S1 to S5
Legends for Movies S1 to S10
SI References

Other supplementary materials for this manuscript include the following:

Movies S1 to S10

Supporting Information Text

1. Defect tracking

Determination of the efficacy of the various control methods proposed in this work hinges upon the ability to track the positions and orientations of topological defects in the simulated nematic texture. This was accomplished through the use of a custom Matlab program employing an algorithm which we now describe.

Given a nematic texture at some moment in time specified by $\mathbf{Q}$, we first calculate the director phase angle θ at each grid point:

$$\theta(x,y) = \frac{1}{2}\arctan\left(\frac{Q_{xy}(x,y)}{Q_{xx}(x,y)}\right) \mod \pi. \qquad \text{[S1]}$$

Next, we must consider closed loops on each small square of four neighboring grid points, to determine whether a path integral over the nematic phase angle has a nonzero topological strength. Let (x, y) denote the position of the bottom right grid point of such a square. Then, we define the following function to calculate approximately differential changes in angles:

$$\Delta\theta(x_1,y_1;x_2,y_2) \equiv \begin{cases} \theta(x_2,y_2)-\theta(x_1,y_1)-\pi, & \text{if } \theta(x_2,y_2)-\theta(x_1,y_1) \geq \pi/2 \\ \theta(x_2,y_2)-\theta(x_1,y_1)+\pi, & \text{if } \theta(x_2,y_2)-\theta(x_1,y_1) \leq -\pi/2 \\ \theta(x_2,y_2)-\theta(x_1,y_1), & \text{otherwise.} \end{cases} \qquad \text{[S2]}$$

The various cases in the above definition are to account for the $\mathbb{Z}_2$ symmetry of the nematic, ensuring that the angle differences used are the smallest in magnitude of their multiple equivalent values. We then calculate the total phase rotation $\Phi(x, y)$ as one circles this square loop:

$$\begin{aligned}\Phi(x,y) = {} & \Delta\theta(x,y;x,y+\Delta x) + \Delta\theta(x,y+\Delta x;x-\Delta x,y+\Delta x) \\ & + \Delta\theta(x-\Delta x,y+\Delta x;x-\Delta x,y) + \Delta\theta(x-\Delta x,y;x,y).\end{aligned} \qquad \text{[S3]}$$

Given how we've defined our function $\Delta\theta$ to take care of the multiple values of θ, the total phase rotation Φ turns out to be related to the previously defined topological strength by $\Phi = -2\pi\nu$. Therefore, if $\Phi(x, y) \approx -\pi$ within a numerical tolerance of 0.3π, then we have found that the square loop whose bottom right point is located at (x, y) encircles the center of a strength $+1/2$ topological defect. Similarly, $\Phi(x, y) \approx \pi$ corresponds to encircling a strength $-1/2$ defect. Finally, to track the locations of defect cores, we introduce the logical matrices $\mathcal{L}_{+1/2}$ and $\mathcal{L}_{-1/2}$:

$$\mathcal{L}_{\pm 1/2}(x,y) \equiv \begin{cases} 1, & \text{if } |(\Phi(x,y)/\pi) \pm 1| < 0.3 \\ 0, & \text{otherwise.} \end{cases} \qquad \text{[S4]}$$

For the purpose of calculating and plotting defect locations, we thus know that if $\mathcal{L}_{\pm 1/2}(x, y) = 1$, there must be a $\pm 1/2$ defect located at $(x - \frac{\Delta x}{2}, y + \frac{\Delta x}{2})$. This is insufficient information to fully describe the defect gas state, however, as we also need to know the polarization of $\pm 1/2$ defects. Towards this end, we define the loop average $\langle f(x, y)\rangle_{\mathcal{L}}$ of some function $f(x, y)$ as follows:

$$\langle f(x,y)\rangle_{\mathcal{L}} \equiv \frac{1}{4}\left[f(x,y) + f(x,y+\Delta x) + f(x-\Delta x,y+\Delta x) + f(x-\Delta x,y)\right] \qquad \text{[S5]}$$

Then, we calculate the angle ψ^ν that each $\nu = \pm 1/2$ defect is rotated from the positive x-axis using an expression derived in Ref. (S1):

$$\psi^\nu(x,y) = \mathcal{L}_\nu(x,y)\left(\frac{\nu}{1-\nu}\right)\tan^{-1}\left[\frac{\langle \text{sgn}(\nu)\partial_x Q_{xy} - \partial_y Q_{xx}\rangle_{\mathcal{L}}}{\langle \partial_x Q_{xx} + \text{sgn}(\nu)\partial_y Q_{xx}\rangle_{\mathcal{L}}}\right]. \qquad \text{[S6]}$$

The logical matrices $\mathcal{L}_\nu$ ensure that polarizations are only calculated for loops containing defects.

2. Numerical implementation of control protocols

A. Active topological tweezer protocols. All of the tweezer procedures we have demonstrated consist of a series of discrete steps, during which each tweezer moves along a straight-line path at a constant speed. This is done by applying an activity pattern with respect to a comoving coordinate system, defined by

$$\mathbf{r}_T \equiv \mathbf{r} - [\mathbf{r}_0 + \mathbf{V}(t - t_{\text{step}})], \qquad \text{[S7]}$$

where $\mathbf{r}$ is the position vector in the lab frame, t_{step} is the start time of the current step, $\mathbf{r}_0$ is the initial position of the tweezer's center at the beginning of the step, and $\mathbf{V}$ is the constant velocity vector with which the tweezer moves during that step. At the end of each step, $\mathbf{r}_0$ is redefined, generally to be the tweezer's final position at the end of the previous step, unless otherwise specified. The velocity vector $\mathbf{V}$ is also changed with each new step to redirect the tweezer, or set to zero to hold it still.

All tweezer activity patterns are based off of the profile (smoothed version of Eq. 5 in Main text)

$$\alpha_{\text{T}}(\mathbf{r}_T) = \frac{\alpha_0}{2}\left[1 + A\sin(2\phi_T) + B\cos(2\phi_T)\right]\left[1 - \tanh\left(\frac{|\mathbf{r}_T| - R}{w/4}\right)\right], \qquad \text{[S8]}$$

where $\phi_T = \arctan(y_T/x_T)$ is the comoving polar coordinate. When $A = B = 0$, this pattern results in a constant activity α_0 within a circle of radius $R - \frac{w}{2}$, zero activity outside of $R + \frac{w}{2}$, and a smooth interface of width w between the two regions. For nonzero A or B, restricted so that $A^2 + B^2 \leq 1$, the result is a 2-fold rotationally symmetric activity profile, which in accordance with the selection rule produces a nonzero net flow at the core of $\pm 1/2$ defects.

A.1. $-1/2$ ***Tweezer Protocol.*** The tweezer procedure depicted in Fig. 2A-C and Movie S1, used to move a $-1/2$ defect along an L-shaped path, was performed over a total time period of 1520 units, with a tweezer of radius $R = 12$ and interface width $w = 2$. The tweezer and defect were both initialized at lab frame coordinates $\mathbf{r}_0 = (-16, -16)$. All other parameters were chosen according to the time-dependent protocol in Table S1

t_{step}	$\mathbf{V}$	α_0	A	B
0.0	$0.05\hat{\mathbf{x}}$	-5	0	1
640.0	$\mathbf{0}$	$-5\cos^2\left(\frac{\pi(t-640)}{80}\right)$	0	1
680.0	$\mathbf{0}$	$-5\cos^2\left(\frac{\pi(t-640)}{80}\right)$	$-\sin(\frac{2\pi}{3})$	$\cos(\frac{2\pi}{3})$
720.0	$\mathbf{0}$	-5	$-\sin(\frac{2\pi}{3})$	$\cos(\frac{2\pi}{3})$
760.0	$0.05\hat{\mathbf{y}}$	-5	$-\sin(\frac{2\pi}{3})$	$\cos(\frac{2\pi}{3})$
1400.0	$\mathbf{0}$	$-5\exp\left(\frac{-(t-1400)}{10}\right)$	$-\sin(\frac{2\pi}{3})$	$\cos(\frac{2\pi}{3})$

Table S1. $-1/2$ **tweezer protocol**

A.2. $+1/2$ ***Tweezer Protocol.*** The tweezer procedure depicted in Fig. 2D-F and Movie S2, used to propel a $+1/2$ defect at an angle along a Λ-shaped path, was performed over a total time period of 1800 units, with a tweezer of radius $R = 12$ and interface width $w = 2$. The tweezer and defect were both initialized at lab frame coordinates $\mathbf{r}_0 = (-20, -10)$. All other parameters were chosen according to the time-dependent protocol given in Table S2.

t_{step}	$\mathbf{V}$	α_0	A	B
0.0	$0.025\hat{\mathbf{x}} + 0.025\hat{\mathbf{y}}$	-0.5	1	0
800.0	$\mathbf{0}$	$-0.5\cos^2\left(\frac{\pi(t-800)}{100}\right)$	1	0
850.0	$\mathbf{0}$	$-0.5\cos^2\left(\frac{\pi(t-800)}{100}\right)$	-1	0
900.0	$0.025\hat{\mathbf{x}} - 0.025\hat{\mathbf{y}}$	-0.5	-1	0
1700.0	$\mathbf{0}$	$-0.5\exp\left(\frac{-(t-1700)}{10}\right)$	-1	0

Table S2. $+1/2$ **tweezer protocol**

A.3. Braiding Protocol. The braiding procedure depicted in Fig. 2G-I and Movie S3 was run for a total of 6500 time units, and was initialized in a defect-free uniform state, with the nematic director aligned along the y-axis. We first impose a low spatially constant activity α_C, over which we apply a mirrored pair of elliptical "ramps" of activity to nucleate two pairs of defects. These "nucleation ramps" were produced with the following activity pattern, where the $\pm$ denotes the pattern applied to the $\pm y$ half of the plane:

$$\alpha_{\text{NR}}(\mathbf{r}_T) = \frac{\alpha_0}{2}\left[1 \pm \alpha_1 x_T\right] \tanh\left(\frac{\sqrt{(\frac{x_T}{2})^2 + y_T^2} - R}{w/4}\right) \qquad \text{[S9]}$$

The ramps were initialized at $x_0 = 0$, $y_0 = \pm 18$, and moved with constant velocity $\mathbf{v}_T = \pm 0.075\hat{\mathbf{x}}$ for the duration of their existence. We kept $R = 4$ and $w = 1$ constant, and set the remaining parameters of this nucleation stage according to the protocol detailed in Table S3.

After creating and separating the defect pairs, we apply two mirrored pairs of tweezers of the form specified in Eq. S8, first to move the $+1/2$ defects out of the way, and then to braid the -1/2 defects around one another. The $+1/2$ tweezers were applied with parameters set by the protocol in Table S4.

The -1/2 tweezers, which perform the actual braiding, follow the protocol given in Table S5

t_{step}	α_C	α_0	α_1
0.0	-2	-10	0.0312
250.0	0	-12	0.0312
325.0	0	$-12\exp\left(\frac{-(t-325)}{10}\right)$	0.0312
350.0	0	0	0

Table S3. Nucleation protocol

t_{step}	$\mathbf{r}_0$	$\mathbf{V}$	α_0	A	B	R	w
0.0	$\mathbf{0}$	$\mathbf{0}$	0	0	0	0	0
250.0	$\pm(-15\hat{\mathbf{x}}+20\hat{\mathbf{y}})$	$\pm(0.0313\hat{\mathbf{x}}+0.0157\hat{\mathbf{y}})$	-2	1	0	9	2
1250.0	$\pm(-47.3\hat{\mathbf{x}}+11\hat{\mathbf{y}})$	$\mathbf{0}$	-1	1	0	6	1
4607.1	$\mathbf{0}$	$\mathbf{0}$	0	0	0	0	0

Table S4. $+1/2$ **braiding protocol**

t_{step}	$\mathbf{r}_0$	$\mathbf{V}$	α_0	A	B	R	w
0.0	$\mathbf{0}$	$\mathbf{0}$	0	0	0	0	0
350.0	$\pm(13\hat{\mathbf{x}}+20\hat{\mathbf{y}})$	$\mp 0.035\hat{\mathbf{y}}$	-5.5	-1	0	9	2
1378.6	$\pm(14\hat{\mathbf{x}}-12\hat{\mathbf{y}})$	$\mp 0.035\hat{\mathbf{x}}$	-5.5	-1	0	9	2
2278.6	$\pm(-18\hat{\mathbf{x}}-12\hat{\mathbf{y}})$	$\pm 0.035\hat{\mathbf{y}}$	-5.5	1	0	9	2
3178.6	$\pm(-18\hat{\mathbf{x}}+18\hat{\mathbf{y}})$	$\pm 0.035\hat{\mathbf{x}}$	-5.5	1	0	9	2
4607.1	$\mathbf{0}$	$\mathbf{0}$	0	0	0	0	0

Table S5. $-1/2$ **braiding protocol**

B. Collective control of active defects. All spatiotemporally averaged data were produced using the following binning scheme: Every 50 timesteps, the nematic texture at each gridpoint was saved, and defect locations and orientations determined using the previously described algorithms. To produce a smooth function of x for each observable (n, ρ, p, & T_3), defect locations and orientations were spatially binned in strips of width $\Delta x = 2$ (i.e., over 4 gridpoints in the x-direction) and spanning the full length of the box in the y-direction, and temporally binned over 20 such datapoints (i.e., over a timespan of 100 time units). These bin sizes were chosen as the smallest possible bins which allowed us to produce smoothed distributions in x. These curves were then averaged over time to produce the means and standard deviations shown in Figures 3-5 in the main text, and used for fitting parameters to data from polynomial activity profiles.

B.1. Activity profiles. Collective spatial patterning of defects (Fig. 3, Movies S4-S5) was achieved using the following profile for an "active strip":

$$\alpha_{\text{strip}}(x) = \frac{\alpha_0}{2}\left[\tanh\left(\frac{x+W_s/2}{w/4}\right) - \tanh\left(\frac{x-W_s/2}{w/4}\right)\right] . \tag{S10}$$

For the dynamic response simulations (Fig. 4, Movies S6-S7), we use the same activity profile (α_{strip}) with the interface width w replaced by $w(t) = w_{\min} + [(w_{\max} - w_{\min})/2](1 - \cos(\omega t))$. For the defect surfing simulations (Fig. 5, Movies S8-10), we translate the active strip at constant speed using $\alpha_{\text{strip}}(x - Vt)$.

3. Analytical derivation of defect dynamics in spatial activity patterns

In this section, we compute the flows generated by topological defects in the presence of spatially varying activity profiles. We provide the details of the additive framework for controlling individual defects and the formulation of the defect hydrodynamic model.

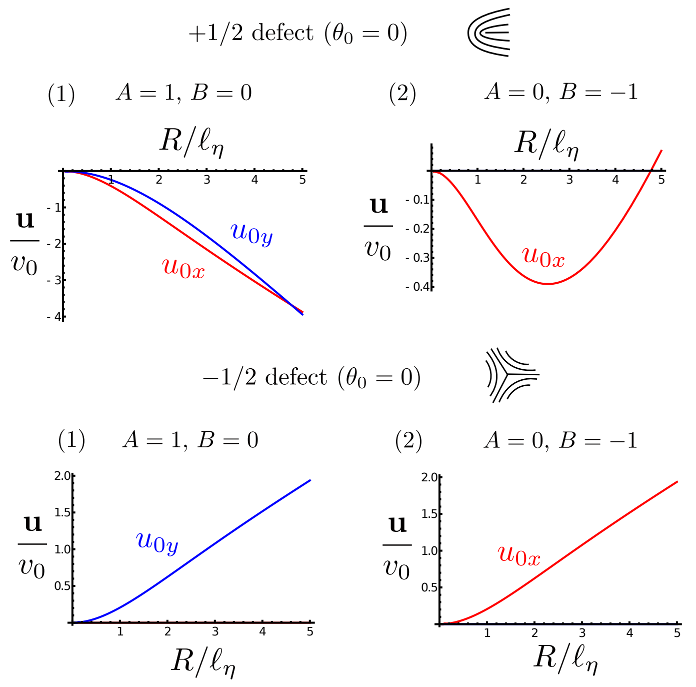

Fig. S1. Flow generated by topological tweezers. The active flow velocity at the defect core ($\mathbf{u}_0$, Eqs. S31, S32; solid curves) is plotted as a function of R/ℓ_η (R: tweezer radius, ℓ_η: screening length) for both $\pm 1/2$ defects using the activity profile given in Eq. 5 (Main text) with different values of A, B. The defect core size is set to $a = 0$ and the defect orientation is taken to be horizontal with $\theta_0 = 0$ (as shown in the schematics). The flow is normalized by the bare self-propulsion speed $v_0 = |\alpha_0|S_0/\Gamma R$ and its x (red) and y (blue) components are separately plotted (components not shown vanish). Nonzero A allows flows along the y-axis for both defects, perpendicular to the defect orientation. Changing the sign of A flips the direction of u_{0y}. Flows along the x-axis depend on B and the screening length ℓ_η. All examples use $A^2 + B^2 = 1$ to obtain maximal effect of activity gradients.

A. Structure and flow of isolated active defects. The 2D orientational order of the active nematic is locally characterized by an alignment tensor $Q_{ij} = S(2\hat{n}_i\hat{n}_j - \delta_{ij})$ with director $\hat{\mathbf{n}} = (\cos\theta, \sin\theta)$. An isolated $\pm 1/2$ defect at the origin is described by $\theta = \pm\phi/2 + \theta_0$, where ϕ is the polar angle and θ_0 dictates the orientation of the defect. The scalar order parameter S is taken to be constant ($S = S_0$) outside the core of the defect ($r \geq a$, a is the core size). The orientation of a $+1/2$ defect is captured by a unit vector (S1, S2)

$$\hat{\mathbf{e}} = \lim_{r\to 0} \frac{\boldsymbol{\nabla}\cdot\mathbf{Q}}{|\boldsymbol{\nabla}\cdot\mathbf{Q}|} = (\cos\psi_+, \sin\psi_+)\ , \quad \psi_+ = 2\theta_0\ . \qquad \text{[S11]}$$

On the other hand, the orientation of the three-fold symmetric $-1/2$ defect is more complicated and described by a rank three symmetric tensor Θ_{ijk} given by (S2)

$$\Theta_{ijk} = \lim_{r\to 0} \frac{\langle \partial_i Q_{jk} + \partial_j Q_{ik} + \partial_k Q_{ij}\rangle}{3|\langle \partial_k Q_{ij}\rangle|} = \hat{t}_i\hat{t}_j\hat{t}_k - \frac{1}{4}\left[\delta_{ij}\hat{t}_k + \delta_{kj}\hat{t}_i + \delta_{ik}\hat{t}_j\right]\ , \qquad \text{[S12]}$$

$$\hat{\mathbf{t}} = (\cos\psi_-, \sin\psi_-)\ , \quad \psi_- = \frac{2\theta_0}{3}\ , \qquad \text{[S13]}$$

where $\langle\cdot\rangle$ is an angular average around the defect core. One can check that Θ_{ijk} vanishes if any two indices are contracted. As a result, Θ_{ijk} only has two nonzero components ($\Theta_{xxx} = -\Theta_{xyy} = (1/4)\cos 3\psi_-$, $\Theta_{yyy} = -\Theta_{xxy} = -(1/4)\sin 3\psi_-$), and an alternate representation that we will use interchangeably is the triatic complex parameter $\Theta_3 = e^{3i\psi_-}$.

The flow generated by the defect is computed using Stokes equation (Eq. S14). As the nematic texture of an isolated defect is an equilibrium solution, both the molecular field and the elastic stress vanishes, i.e., $\mathbf{H} = \mathbf{0}$ and $\boldsymbol{\sigma}^{el} = \mathbf{0}$. Stokes equation then simplifies and the flow generated by the active stress $\boldsymbol{\sigma}^a = \alpha\mathbf{Q}$ is computed from

$$\ell_\eta^2\nabla^2\mathbf{u} - \mathbf{u} + \frac{1}{\Gamma}\boldsymbol{\nabla}\cdot\boldsymbol{\sigma}^a - \frac{1}{\Gamma}\boldsymbol{\nabla}\Pi = 0\ , \quad \boldsymbol{\nabla}\cdot\mathbf{u} = 0\ , \qquad \text{[S14]}$$

where we have used the hydrodynamic screening length $\ell_\eta = \sqrt{\eta/\Gamma}$. Incompressibility is enforced by using a 2D stream function, $\mathbf{u} = -\hat{\mathbf{z}}\times\boldsymbol{\nabla}\Psi$ ($u_x = \partial_y\Psi\, , u_y = -\partial_x\Psi$), so the vorticity $\omega = \hat{\mathbf{z}}\cdot(\boldsymbol{\nabla}\times\mathbf{u}) = -\nabla^2\Psi$ satisfies

$$\ell_\eta^2\nabla^2\omega - \omega + \frac{1}{\Gamma}\mathcal{W} = 0\ , \qquad \text{[S15]}$$

where $\mathcal{W} = \hat{\mathbf{z}}\cdot(\boldsymbol{\nabla}\times\boldsymbol{\nabla}\cdot\boldsymbol{\sigma}^a)$ is the rotational component of the active force density. For a $\pm 1/2$ defect, we will denote the corresponding stream-function as $\Psi^\pm$, the flow as $\mathbf{u}^\pm$ and the vorticity as $\omega^\pm$.

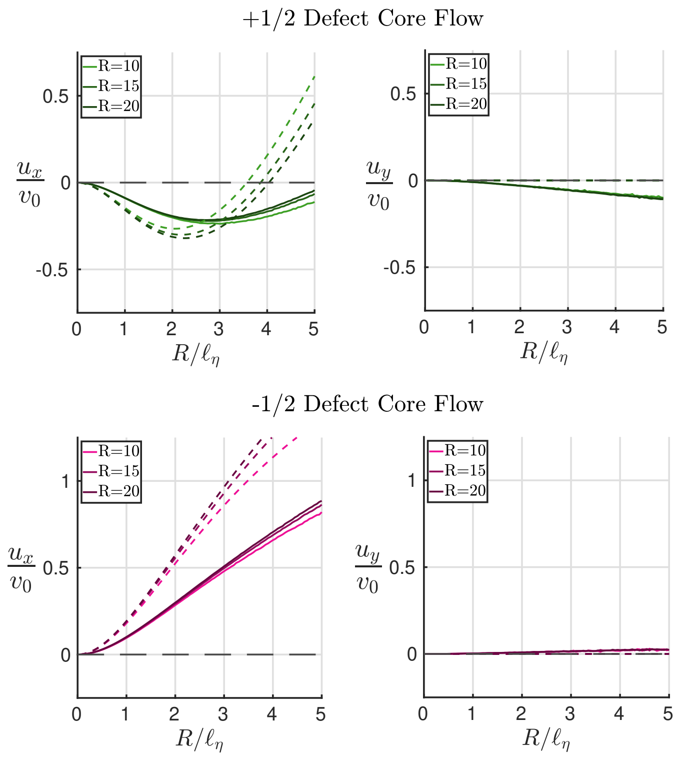

Fig. S2. Comparing numerical and theoretical flows generated by topological tweezers. The active flow velocity at the defect core ($\mathbf{u}_0$) is numerically computed for a $+1/2$ (top) and $-1/2$ (bottom) defect by solving Stokes' equation (Eq. 2, Main text) in a periodic box of size $2L = 128$ with a $\mathbf{Q}$ tensor corresponding to an ideal isolated defect with $\theta_0 = 0$ and an activity pattern for a tweezer (Eq. 5, Main text) with $\alpha_0 = -1$, $A = 0$ and $B = -1$. The tweezer radius (R) and screening length (ℓ_η) are separately varied as shown in the legend. The angle averaged flow velocity (x-component: left, y-component: right) at the origin is nondimensionalized by $v_0 = |\alpha_0|S_0/\Gamma R$ and plotted for both the numerical solution (solid curves) and the analytical prediction (Eqs. S31, S32 with core size $a = 1$; dashed curves). For both defects, u_y is theoretically predicted to be zero, but the numerical solution obtains a small but nonvanishing transverse flow. The numerical curves for u_x are similarly qualitatively consistent with the theory prediction. We attribute the quantitative discrepancy between the curves to the periodic boundary conditions and details of the defect core that are neglected in the theory calculation.

Defects behave like quasiparticles driven by self-generated active flows at their core (S3, S4). Following similar analysis previously performed for the case of both homogeneous activity (S3, S5–S7) and simple gradient profiles (S8), we compute the active flow and vorticity near the core of a $\pm1/2$ defect defined as

$$\mathbf{u}_0^{\pm} = \lim_{r\to a}\int_0^{2\pi}\frac{\mathrm{d}\phi}{2\pi}\mathbf{u}^{\pm}(r,\phi) = \int_0^{2\pi}\frac{\mathrm{d}\phi}{2\pi}\left(\sin\phi, -\cos\phi\right)\left(\partial_r\Psi^{\pm} + \frac{\Psi^{\pm}}{r}\right)\Bigg|_{r=a}\ , \tag{S16}$$

$$\omega_0^{\pm} = \lim_{r\to a}\int_0^{2\pi}\frac{\mathrm{d}\phi}{2\pi}\ \omega^{\pm}(r,\phi)\ , \tag{S17}$$

where we have integrated by parts and used the fact that $u_x = (1/r)\cos\phi\partial_\phi\Psi + \sin\phi\partial_r\Psi$ and $u_y = (1/r)\sin\phi\partial_\phi\Psi - \cos\phi\partial_r\Psi$. From Eqs. S16, S17 we directly see that only the zeroth angular harmonic (i.e., constant in ϕ) of $\omega^{\pm}$ (or equivalently $\Psi^{\pm}$) contributes to the defect rotation rate $\omega_0^{\pm}$, and only the first angular harmonic of $\omega^{\pm}$ and $\Psi^{\pm}$ contributes to the defect velocity $\mathbf{u}_0^{\pm}$.

B. General proof of the selection rule. Here we provide a proof for the symmetry-based selection rule that underlies the additive control framework and the construction of active topological tweezers.

An arbitrary activity pattern $\alpha(\mathbf{r})$ can be expanded in an angular Fourier basis,

$$\alpha(\mathbf{r}) = \sum_{n=-\infty}^{\infty}\tilde{\alpha}_n(r)\ e^{in\phi}\ , \tag{S18}$$

where ϕ is polar angle and $\tilde{\alpha}_n(r)$ are complex functions ($\tilde{\alpha}_n^* = \tilde{\alpha}_{-n}$) of the distance r from the defect core. Note that n characterizes the (n-fold) rotational symmetry of the activity profile. We can similarly expand the stream-function and vorticity in an angular Fourier basis as

$$\Psi(\mathbf{r}) = \sum_{n=-\infty}^{\infty}\tilde{\Psi}_n(r)\ e^{in\phi}\ , \quad \omega(\mathbf{r}) = \sum_{n=-\infty}^{\infty}\tilde{\omega}_n(r)\ e^{in\phi}\ . \tag{S19}$$

We shall now prove the following general theorem whose corollary is the desired selection rule.

 Suraj Shankar, Luca V. D. Scharrer, Mark J. Bowick, M. Cristina Marchetti

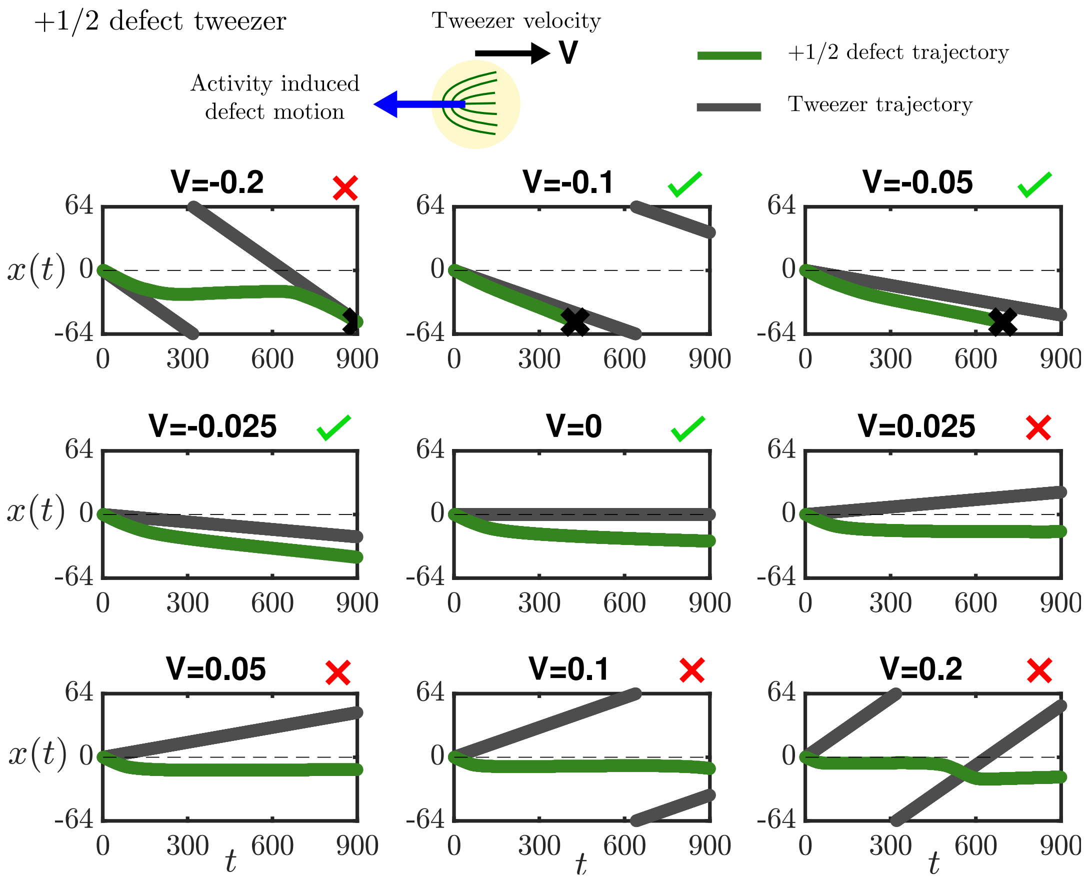

Fig. S3. Characterizing the $+1/2$ topological tweezer. A tweezer of size $R = 12$ and activity profile in Eq. 5 (Main text) with parameters $\alpha_0 = -1.5$, $A = 0$, $B = 0$ is chosen to move a $+1/2$ defect ($\theta_0 = 0$). The screening length $\ell_\eta = 5$. The activity induced defect motion is in the $-\hat{x}$ direction (speed ~ 0.1) and the tweezer disc is itself moved along the x-axis with a velocity V ($V > 0$: right moving, $V < 0$: left moving). The defect tracks (green line) the tweezer motion (gray line) when the tweezer speed is smaller or equal to the activity induced motion ($-0.1 \lesssim V \leq 0$, panels marked by green ticks). When the tweezer moves too fast ($V < -0.1$) or moves in the opposite direction to the active motion ($V > 0$), the defect quickly leaves the active region of the tweezer (after a time $\sim R/V$) and fails to track the tweezer motion (panels marked by red crosses). In some cases, the tweezer trajectory wraps around due to periodic boundary conditions and causes a small displacement of the defect when the tweezer passes over. Black crosses terminating some defect trajectories represent points where the $+1/2$ defect annihilated with a $-1/2$ defect present at the same location.

Theorem 3.1. *Consider an isolated defect with topological charge ν and flow $\mathbf{u}$ satisfying linear Stokes equation (Eq. S14). Suppose the flow has $\tilde{\Psi}_m(r) \neq 0$ for some $m \in \mathbb{Z}$, then $\exists\ \tilde{\alpha}_n(r) \neq 0$ that generates $\tilde{\Psi}_m(r)$, if and only if $n \in \mathbb{Z}$ satisfies the equation $|m| = |2 - n - 2\nu|$.*

Proof. As Stokes equation (Eq. S14) is linear, we can perform an angular Fourier transform and the different Fourier modes decouple. Eq. S15 simplifies to give,

$$\ell_\eta^2 \left[\tilde{\omega}_n'' + \frac{1}{r}\tilde{\omega}_n' - \frac{n^2}{r^2}\tilde{\omega}_n \right] - \tilde{\omega}_n + \frac{1}{\Gamma}\tilde{\mathcal{W}}_n = 0 \,, \qquad \text{[S20]}$$

$$\tilde{\Psi}_n'' + \frac{1}{r}\tilde{\Psi}_n' - \frac{n^2}{r^2}\tilde{\Psi}_n = -\tilde{\omega}_n \,. \qquad \text{[S21]}$$

It is convenient to write the curl of the active force density $\mathcal{W} = \hat{\mathbf{z}} \cdot (\boldsymbol{\nabla} \times \boldsymbol{\nabla} \cdot \boldsymbol{\sigma}^a)$ in complex form as follows

$$\mathcal{W} = \mathrm{Re}\left[-i\left(\partial_x^2 - \partial_y^2 - 2i\partial_x\partial_y\right)\left(\sigma_{xx}^a + i\sigma_{xy}^a\right)\right] \,. \qquad \text{[S22]}$$

Using the fact that $Q_{xx} + iQ_{xy} = S_0 e^{2i(\nu\phi+\theta_0)}$ for an isolated defect texture with charge ν, we have for a spatially varying α (Eq. S18)

$$\sigma_{xx}^a + i\sigma_{xy}^a = S_0 \sum_{n=-\infty}^{\infty} \tilde{\alpha}_n(r)\, e^{i[(2\nu+n)\phi+2\theta_0]} \,. \qquad \text{[S23]}$$

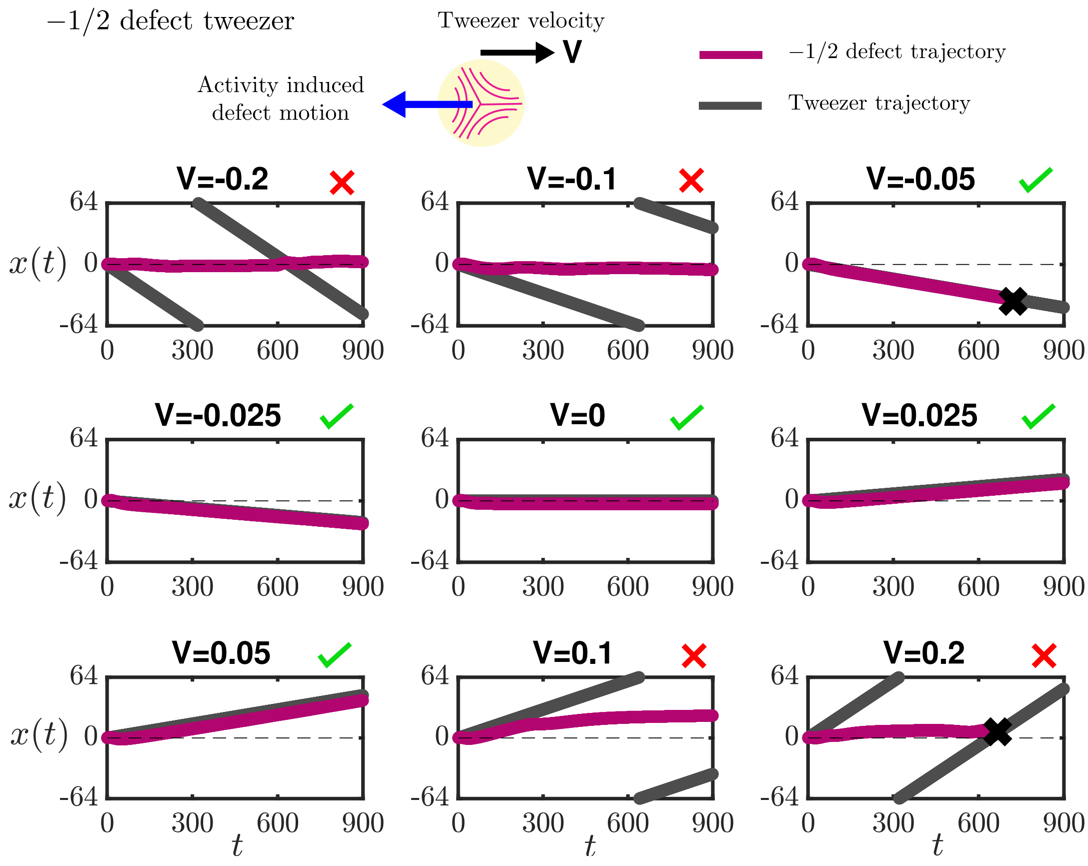

Fig. S4. Characterizing the $-1/2$ topological tweezer. A tweezer of size $R = 12$ and activity profile in Eq. 5 (Main text) with parameters $\alpha_0 = -5$, $A = 0$, $B = -1$ is chosen to move a $-1/2$ defect ($\theta_0 = 0$). The screening length $\ell_\eta = 5$. The activity induced defect motion is in the $-\hat{x}$ direction (speed ~ 0.05) and the tweezer disc is itself moved along the x-axis with a velocity V ($V > 0$: right moving, $V < 0$: left moving). The defect tracks (magenta line) the tweezer motion (gray line) when the tweezer speed is smaller or equal to the activity induced motion ($|V| \lesssim 0.05$, panels marked by green ticks). When the tweezer moves too fast in either direction ($|V| > 0.05$), the defect quickly leaves the active region of the tweezer (after a time $\sim R/V$) and fails to track the tweezer motion (panels marked by red crosses). Unlike the $+1/2$ tweezer shown in Extended Data Fig. S3, the $-1/2$ defect can be dragged by an active tweezer even in a direction opposite to its activity induced motion, as long as the tweezer speed is small enough. In some cases, the tweezer trajectory wraps around due to periodic boundary conditions and causes a small displacement of the defect when the tweezer passes over. Black crosses terminating some defect trajectories represent points where the $-1/2$ defect annihilated with a $+1/2$ defect present at the same location.

From Eq. S22, we then obtain the angular Fourier mode $\tilde{\mathcal{W}}_m(r) = \int_0^{2\pi} (\mathrm{d}\phi/2\pi)\, e^{-im\phi} \mathcal{W}(\mathbf{r})$ to be

$$\tilde{\mathcal{W}}_m = i\frac{S_0}{2}\left[-e^{2i\theta_0}\left(\tilde{\alpha}''_n + \frac{(3+2m)}{r}\tilde{\alpha}'_n + \frac{m(m+2)}{r^2}\tilde{\alpha}_n\right)\delta_{m,-2+n+2\nu}\right.$$
$$\left. + e^{-2i\theta_0}\left(\tilde{\alpha}''_{-n} + \frac{(3-2m)}{r}\tilde{\alpha}'_{-n} + \frac{m(m-2)}{r^2}\tilde{\alpha}_{-n}\right)\delta_{m,2-n-2\nu}\right] . \quad \text{[S24]}$$

From Eq. S24, we immediately see that $\tilde{\mathcal{W}}_m \neq 0$ only for $m = \pm(2 - n - 2\nu)$. By linearity of Stokes equation (Eq. S20, S21), $\tilde{\Psi}_m \neq 0 \iff \tilde{\mathcal{W}}_m \neq 0$ and from Eq. S24, $\tilde{\mathcal{W}}_m \neq 0 \iff \tilde{\alpha}_n \neq 0$ for some $\tilde{\alpha}_n(r)$ with $|m| = |2 - n - 2\nu|$. This concludes the proof. □

We now use Theorem 3.1 to obtain the selection rule. In terms of the angular Fourier moments, the angle averaged flow and vorticity at the defect core (Eqs. S16, S17) simplify to

$$u_{0x} + iu_{0y} = -i\left(\tilde{\Psi}'_{-1} + \frac{1}{r}\tilde{\Psi}_{-1}\right)\bigg|_{r=a} , \quad \omega_0 = \tilde{\omega}_0|_{r=a} . \quad \text{[S25]}$$

As we are interested in the translations and rotations of the defect, we only need to focus on and compute $\tilde{\Psi}_0$ and $\tilde{\Psi}_{-1}$ in terms of the α to obtain the active flow and vorticity at the defect core. So we only consider the cases $|m| = 1$ and $|m| = 0$ in the Theorem 3.1 and obtain the required selection rule.

To simplify notation, it is more transparent and easier to use the symmetry of the defect rather than the charge. For a 2D nematic disclination with topological charge ν, the rotational symmetry (s) of the defect texture is related to the charge by (S2)

$$s = 2|1 - \nu| . \quad \text{[S26]}$$

As expected, a $\nu = +1/2$ defect is polar or 1-fold symmetric ($s = 1$), a $\nu = -1/2$ defect is 3-fold symmetric ($s = 3$), a $\nu = +1$ vortex is isotropic ($s = 0$) and a $\nu = -1$ antivortex is 4-fold symmetric ($s = 4$).

Combining Theorem 3.1 with Eqs. S25, S26, we can finally state the symmetry selection rule in compact form as follows.

Symmetry selection rule: Consider an isolated defect in 2D with topological charge ν and rotational symmetry $s > 0$ subject to an activity pattern $\alpha(\mathbf{r})$ with an n-fold symmetric component, i.e., $\tilde{\alpha}_{\pm n}(r) \neq 0$ ($n > 0$). Then a necessary condition for

- activity-induced flow velocity at the core ($\mathbf{u}_0 \neq \mathbf{0}$) is $|n - s| = 1$, and
- activity-induced vorticity at the core ($\omega_0 \neq 0$) is $|n - s| = 0$.

For this condition to be sufficient as well, the radial profile of $\tilde{\alpha}_n(r)$ must not be homogeneous solution of Eq. S24.

C. Topological tweezer construction. Here we sketch the design rules used to construct the topological tweezers and analytical characterize their properties. As mentioned in the Main text, there are three main constraints to satisfy - (i) the selection rule for the symmetry of the activity pattern, (ii) the maximum activity is smaller than the bend-instability threshold and (iii) α does not change sign (so the system never switches from extensile to contractile or vice-versa). We choose radially constant activity profiles to minimize gradients and actuation effort and choose the smallest angular variation required to satisfy the symmetry rule. These design principles allow the construction of simple topological tweezers for translating $\pm 1/2$ defects.

To translate either $\pm 1/2$ defect, the symmetry rule dictates that we need atleast an $n = 2$-fold symmetric activity pattern. For a $+1/2$ defect, even the isotropic ($n = 0$) activity pattern will generate propulsion. As mentioned in the Main text, a simple choice for α (centered on the defect) is then

$$\alpha(\mathbf{r}) = \begin{cases} \alpha_0[1 + A\sin(2\phi) + B\cos(2\phi)] & (r \leq R) \\ 0 \quad (r > R) \end{cases} , \qquad \text{[S27]}$$

so $\tilde{\alpha}_0 = \alpha_0$ and $\tilde{\alpha}_{\pm 2} = (\alpha_0/2)(B \mp iA)$. $\alpha_0 < 0$ dictates the maximum extensile activity and $\sqrt{A^2 + B^2} \leq 1$ to maintain fixed sign of α.

The maximal activity $|\alpha_0|$ is chosen to be below the bend-instability threshold. While a detailed calculation of the instability threshold in the tweezer geometry is unavailable, we can estimate the instability threshold in two simple limits. In an infinite system with homogeneous activity, substrate friction Γ alone provides a maximal instability threshold $|\alpha_c| = 2K\Gamma/\gamma(1 + \lambda)$ (S9, S10). In contrast, in the purely viscous limit ($\Gamma = 0$), a finite system of size L also has a bend instability threshold given by $|\alpha_c| = (2K\eta/\gamma(1 + \lambda))(2\pi/L)^2$ (S11, S12). Assuming the tweezer behaves like a finite system of size $L = 2R$, we can approximate a conservative estimate of the instability threshold to be the smallest $|\alpha_c|$ from either limit, i.e., $|\alpha_0| \lesssim [2K\Gamma/\gamma(1 + \lambda)]\min(1, (\pi\ell_\eta/R)^2)$, which along with $\lambda \sim 1$ gives the expression in the main text.

To compute the active flow generated by either $\pm 1/2$ defect, we have to solve Eq. S14 in the plane with appropriate far-field boundary conditions ($|\mathbf{u}| \to 0$ as $|\mathbf{r}| \to \infty$). As α is discontinuous across $r = R$ (the tweezer boundary, see Eq. S27), the vorticity-streamfunction approach adopted above is technically cumbersome as the active vorticity source $\mathcal{W}$ is highly singular at $r = R$ (see Eq. S24). We instead adopt a simpler solution by using the Green's function for Eq. S14 to write $\mathbf{u}(\mathbf{r}) = \int \mathrm{d}\mathbf{r}' \mathbf{G}(\mathbf{r} - \mathbf{r}') \cdot [\boldsymbol{\nabla}' \cdot \boldsymbol{\sigma}^a(\mathbf{r}')]$, where (S13)

$$G_{ij}(\mathbf{r}) = \frac{1}{2\pi\eta}\left[G_1\left(\frac{r}{\ell_\eta}\right)\delta_{ij} + \frac{r_i r_j}{r^2}G_2\left(\frac{r}{\ell_\eta}\right)\right] , \qquad \text{[S28]}$$

$$G_1(z) = -\frac{1}{z^2} + \frac{1}{2}[K_0(z) + K_2(z)] , \qquad \text{[S29]}$$

$$G_2(z) = \frac{2}{z^2} - K_2(z) , \qquad \text{[S30]}$$

where $K_n(z)$ are modified Bessel functions of the second kind.

The flow velocity at the core is then obtained by integrating by parts and setting $\mathbf{r} = \mathbf{0}$, which gives $u_i(\mathbf{0}) = -\int \mathrm{d}\mathbf{r}'\ \partial'_k G_{ij}(\mathbf{r}')\sigma^a_{jk}(\mathbf{r}')$. The integral can be easily performed as $\boldsymbol{\sigma}^a$ is nonsingular everywhere. Upon simplifying, we obtain the core velocity for both $\nu = \pm 1/2$ defects to be

$$\mathbf{u}_0^+ = \frac{v_0}{4}\Big((2f_1 + Bf_2)\cos 2\theta_0 + Af_2\sin 2\theta_0, Af_2\cos 2\theta_0 + (2f_1 - Bf_2)\sin 2\theta_0\Big) , \qquad \text{[S31]}$$

$$\mathbf{u}_0^- = \frac{v_0}{4}f_1\Big(B\cos 2\theta_0 + A\sin 2\theta_0\ , -A\cos 2\theta_0 + B\sin 2\theta_0\Big) , \qquad \text{[S32]}$$

where the overall self-propulsion speed is given by $v_0 = |\alpha_0|S_0/\Gamma R$ (for $\alpha_0 < 0$) and the constants $f_{1,2}$ are given by

$$f_1 = -\frac{R}{\ell_\eta}\int_{a/\ell_\eta}^{R/\ell_\eta} \mathrm{d}z\ zK_1(z) , \qquad \text{[S33]}$$

$$f_2 = \frac{R}{\ell_\eta}\int_{a/\ell_\eta}^{R/\ell_\eta} \mathrm{d}z\left[-\frac{8}{z^2} + zK_3(z)\right] . \qquad \text{[S34]}$$

Here a is the finite defect core size. Note, $f_{1,2}$ remain finite in the $a/\ell_\eta \to 0$ limit. In the opposite (friction dominated) limit where $\ell_\eta \to 0$, the defect core size ($a > 0$) must be retained as a short-distance cutoff.

D. Defect velocity and vorticity for polynomial activity. Here we consider a special case of the more general result in Sec. B by focusing on polynomial activity profiles. In the vicinity of a defect (assumed at the origin), we Taylor expand the activity profile in powers of the distance $\mathbf{r}$ from the core as

$$\alpha(\mathbf{r}) = \alpha_0 + \alpha_i r_i + \frac{1}{2}\alpha_{ij} r_i r_j + \frac{1}{6}\alpha_{ijk} r_i r_j r_k + \mathcal{O}(r^4) , \quad \text{[S35]}$$

where $\alpha_0 = \alpha(\mathbf{0})$, $\alpha_i = \partial_i \alpha(\mathbf{0})$, and so on. In terms of the angular Fourier moments (Eq. S18), we have the following nonvanishing terms

$$\tilde{\alpha}_0(r) = \alpha_0 + \frac{r^2}{4}\alpha_{kk} + \mathcal{O}(r^4) , \quad \text{[S36]}$$

$$\tilde{\alpha}_1(r) = \frac{r}{2}(\alpha_x - i\alpha_y) + \frac{r^3}{16}\left[\alpha_{xxx} + \alpha_{xyy} - i\left(\alpha_{xxy} + \alpha_{yyy}\right)\right] + \mathcal{O}(r^5) , \quad \text{[S37]}$$

$$\tilde{\alpha}_2(r) = \frac{r^2}{8}\left(\alpha_{xx} - \alpha_{yy} - 2i\alpha_{xy}\right) + \mathcal{O}(r^4) , \quad \text{[S38]}$$

$$\tilde{\alpha}_3(r) = \frac{r^3}{48}\left[\alpha_{xxx} - 3\alpha_{xyy} + i\left(\alpha_{yyy} - 3\alpha_{xxy}\right)\right] + \mathcal{O}(r^5) . \quad \text{[S39]}$$

Solving Eq. S24 with $m = 0, 1$ for both $\nu = \pm 1/2$ defects, we obtain (to lowest nontrivial order in activity gradients)

$$\tilde{\mathcal{W}}_0^+ = \frac{3S_0}{2r}\alpha_i \epsilon_{ij}\hat{e}_j + \mathcal{O}(r) , \quad \text{[S40]}$$

$$\tilde{\mathcal{W}}_1^+ = -i\frac{S_0}{2}\left(-\frac{\alpha_0}{r^2} + \frac{3}{4}\alpha_{kk}\right)e^{-2i\theta_0} - i\frac{15S_0}{16}\left(\alpha_{xx} - \alpha_{yy} - 2i\alpha_{xy}\right)e^{2i\theta_0} + \mathcal{O}(r^2) , \quad \text{[S41]}$$

$$\tilde{\mathcal{W}}_0^- = -r\frac{5S_0}{4}\Theta_{ijk}\epsilon_{k\ell}\alpha_{ij\ell} + \mathcal{O}(r^3) , \quad \text{[S42]}$$

$$\tilde{\mathcal{W}}_1^- = \frac{3S_0}{4}\left[\Theta_{yjk}\alpha_{jk} + i\Theta_{xjk}\alpha_{jk}\right] + \mathcal{O}(r^2) , \quad \text{[S43]}$$

where ϵ_{ij} is the antisymmetric Levi-Civita tensor and we have used the $+1/2$ defect polarization $\hat{\mathbf{e}} = (\cos 2\theta_0, \sin 2\theta_0)$ along with the $-1/2$ defect triatic parameter Θ_{ijk}.

To solve for the stream function Ψ and the flow $\mathbf{u}$, we need to specify boundary conditions. As we have Taylor expanded α, we only solve Eq. S15 in a finite circular domain of radius R and enforce no-slip boundary conditions along the boundary, i.e., $\mathbf{u}|_{r=R} = \mathbf{0}$. For simplicity, we work in the friction dominated regime ($\ell_\eta \to 0$) and neglect viscous dissipation (finite ℓ_η only changes things quantitatively as shown in Sec. C). In this limit, Eq. S15 simplifies to $\omega = \mathcal{W}/\Gamma$. Solving for the stream function and setting $\partial_\phi \Psi|_{r=R} = \partial_r \Psi|_{r=R} = 0$, we obtain for the $n = -1$ angular harmonic mode,

$$\tilde{\Psi}_{-1}^+(r) = -i\frac{\alpha_0 S_0 R}{4r\Gamma}\left(1 - \frac{r}{R}\right)^2 e^{2i\theta_0} - \mathcal{A}\frac{S_0 R^3}{6r\Gamma}\left(1 + \frac{2r}{R}\right)\left(1 - \frac{r}{R}\right)^2 , \quad \text{[S44]}$$

$$\tilde{\Psi}_{-1}^-(r) = \frac{3S_0}{4\Gamma}\left[\frac{r^2}{3} - \frac{Rr}{2} + \frac{R^3}{6r}\right]\left(-\Theta_{yjk}\alpha_{jk} + i\Theta_{xjk}\alpha_{jk}\right) , \quad \text{[S45]}$$

where $\mathcal{A} = (-3i/16)[2\alpha_{kk}e^{2i\theta_0} - 5(\alpha_{xx} - \alpha_{yy})e^{-2i\theta_0} - 10i\alpha_{xy}e^{-2i\theta_0}]$. The angularly averaged vorticity is simply given by $\tilde{\mathcal{W}}_0$ in the $\ell_\eta \to 0$ limit, so we have

$$\tilde{\omega}_0^+(r) = \frac{3S_0}{2\Gamma r}\alpha_i\epsilon_{ij}\hat{e}_j , \quad \text{[S46]}$$

$$\tilde{\omega}_0^-(r) = -r\frac{5S_0}{4\Gamma}\Theta_{ijk}\epsilon_{k\ell}\alpha_{ij\ell} . \quad \text{[S47]}$$

Using these solutions for $\omega_0^\pm$ and $\Psi_{-1}^\pm$, we then obtain $\mathbf{u}_0^\pm$ and $\omega_0^\pm$ to be (assuming $R \gg a$)

$$\mathbf{u}_0^+ = \frac{1}{2a\Gamma}\left(\alpha - \frac{21}{8}aR\nabla^2\alpha\right)\hat{\mathbf{e}} + \frac{15R}{8\Gamma}\boldsymbol{\nabla}\boldsymbol{\nabla}\alpha\cdot\hat{\mathbf{e}} , \quad \text{[S48]}$$

$$\mathbf{u}_0^- = -\frac{3R}{2\Gamma}\boldsymbol{\Theta} : \boldsymbol{\nabla}\boldsymbol{\nabla}\alpha = -\frac{3R}{2\Gamma}\left(\mathrm{Re}[\Theta_3\partial^2\alpha], \mathrm{Im}[\Theta_3\partial^2\alpha]\right) , \quad \text{[S49]}$$

$$\omega_0^+ = \frac{3}{2a\Gamma}\hat{\mathbf{z}}\cdot(\boldsymbol{\nabla}\alpha\times\hat{\mathbf{e}}) , \quad \text{[S50]}$$

$$\omega_0^- = \frac{5a}{4\Gamma}\partial_i\partial_j\partial_k\alpha\ \epsilon_{k\ell}\ \Theta_{ij\ell} = -\frac{5a}{2\Gamma}\mathrm{Re}[i\Theta_3\partial^3\alpha] . \quad \text{[S51]}$$

Here, we have also used the complex triatic parameter $\Theta_3 = e^{3i\psi_-} = e^{2i\theta_0}$ and the complex Wirtinger derivatives ($\partial = (1/2)(\partial_x - i\partial_y)$ and $\bar{\partial} = (1/2)(\partial_x + i\partial_y)$ such that $\partial z = 1$ and $\bar{\partial} z = 0$ where $z = x + iy$) to simplify some of the expressions.

The above results show that the only effect of a constant activity gradient ($\alpha = \alpha_0 + \boldsymbol{\alpha}_1\cdot\mathbf{r}$) is to endow the $+1/2$ defect with an angular velocity which aligns its polarization with the direction of increasing (decreasing) activity for extensile, $\alpha < 0$

(contractile, $\alpha > 0$) activity, as recently obtained in Ref. (S14). For either sign of activity, the $+1/2$ defect rotates to propel itself towards decreasing activity. Second order gradients in activity renormalize the self-propulsion of $+1/2$ defects and endow the $-1/2$ defect with translational motion. A third order activity gradient is required to get the $-1/2$ defect to rotate.

The order of the polynomial activity gradient required to generate a nonzero velocity or vorticity at the core of a $\pm 1/2$ defect is entirely governed by the symmetry selection rule described in Sec. B. For polynomial profiles, a simple way to understand the result is to note that a nonzero velocity at the core requires to construct a vector out of the geometric properties of the defect and any available activity gradients. The $+1/2$ defect has polar symmetry described a vector $\hat{\mathbf{e}}$, hence it self-propels for constant and quadratic activity (as both $\alpha_0\hat{\mathbf{e}}$ and $\boldsymbol{\nabla}\boldsymbol{\nabla}\alpha\cdot\hat{\mathbf{e}}$ are the lowest order vectors possible). The three-fold symmetry of a $-1/2$ defect on the other hand is described by a rank-3 symmetric tensor $\boldsymbol{\Theta}$ and requires a rank-2 tensor to create a vector (contracting two indices of $\boldsymbol{\Theta}$ is insufficient to create a vector as $\Theta_{iij} = 0$ by construction). Hence, at lowest order, $-1/2$ defects self-propel in nonzero quadratic activity. Similar arguments also underlie the vorticity expressions.

4. Perturbative derivation of mobility relation for active defects

Here we provide a systematic perturbative derivation of a quasiparticle like description of defect motion induced by active flows following previous works (S3, S7), now generalized to activity patterns. In the simplest setting, neglecting flow alignment and working deep in the ordered phase ($S = S_0$), the director angle θ obeys the simple dynamics

$$\partial_t\theta + \mathbf{u}\cdot\boldsymbol{\nabla}\theta - \frac{1}{2}\omega = \frac{K}{\gamma}\nabla^2\theta \ , \qquad \text{[S52]}$$

with elastic constant K and rotational viscosity γ. In the vicinity of a single slowly moving defect with velocity $\mathbf{v}_d$ (valid when $\tau_n\partial_t \ll 1$ and $|\alpha|\gamma/\Gamma K \ll 1$), we can move to the comoving frame ($\partial_t\theta \to \partial_t\theta - \mathbf{v}_d\cdot\boldsymbol{\nabla}\theta$) and perturbatively expand $\theta(\mathbf{r},t) = \theta^{(0)} + \varepsilon\theta^{(1)} + \varepsilon^2\theta^{(2)} + \mathcal{O}(\varepsilon^3)$, where $\varepsilon \ll 1$ is a book-keeping small parameter so that $|\mathbf{v}_d|, \alpha \sim \mathcal{O}(\varepsilon)$. Thus the flow and vorticity generated by active stresses are $|\mathbf{u}|, |\omega| \sim \mathcal{O}(\varepsilon)$. At lowest order ($\mathcal{O}(\varepsilon^0)$), we simply have the equilibrium defect profile satisfying $\nabla^2\theta^{(0)} = 0$, so $\theta^{(0)}(\mathbf{r},t) = \nu\phi + \theta_0(t)$ where $\nu = \pm 1/2$ is the defect charge and θ_0 is the defect orientation angle. At first order in ε, we have

$$\partial_t\theta^{(0)} + (\mathbf{u} - \mathbf{v}_d)\cdot\boldsymbol{\nabla}\theta^{(0)} - \frac{1}{2}\omega = \frac{K}{\gamma}\nabla^2\theta^{(1)} \ . \qquad \text{[S53]}$$

Imposing the Frobenius solvability condition by multiplying Eq. S53 with the rotational and translational eigenmodes of a defect (1 and $\boldsymbol{\nabla}\theta^{(0)}$ respectively), and integrating over space, we obtain

$$\dot{\theta}_0 = \frac{1}{2\pi L_c^2}\int_0^{L_c} \mathrm{d}^2r \left[\omega - 2\nu\frac{u_\phi}{r}\right] \ , \qquad \text{[S54]}$$

$$\frac{\pi}{4}\ln\left(\frac{L_c}{a}\right)(\mathbf{U}_a - \mathbf{v}_d) = \nu\frac{K}{\gamma}\int_0^{2\pi} \mathrm{d}\phi\, r\frac{\hat{\mathbf{e}}_\phi}{r}\hat{\mathbf{e}}_r\cdot\boldsymbol{\nabla}\theta^{(1)} = -\pi\nu\boldsymbol{\epsilon}\cdot\boldsymbol{\nabla}\theta_{\mathrm{ext}} \ , \qquad \text{[S55]}$$

where L_c is a large scale cutoff (set by system size L, mean defect separation $\xi_d \sim 1/\sqrt{n}$ or distortion length scale $K/(\gamma|\mathbf{v}_d|)$ in the wake of a moving defect, which ever is smaller). In any case, we shall consider the defect mobility as a phenomenological constant and set $\mu = 4/[\gamma\ln(L_c/a)]$. We have used the fact that $\boldsymbol{\nabla}\theta^{(1)} \to \boldsymbol{\nabla}\theta_{\mathrm{ext}}$ as $|\mathbf{r}| \to \infty$ along with the following identities

$$\int_a^{L_c} \mathrm{d}^2r\frac{1}{r^2}\hat{\mathbf{e}}_\phi\hat{\mathbf{e}}_\phi = \pi\ln\left(\frac{L_c}{a}\right)\mathbf{I} \ , \quad \int_0^{2\pi}\mathrm{d}\phi\ \hat{\mathbf{e}}_\phi\hat{\mathbf{e}}_r = -\pi\boldsymbol{\epsilon} \ . \qquad \text{[S56]}$$

The active flow induces a defect velocity $\mathbf{U}_a = (1/\pi\ln(L_c/a))\int(\mathrm{d}^2r/r^2)[u_\phi - 2\nu r\omega]\hat{\mathbf{e}}_\phi$ which simplifies to give

$$U_{ax} + iU_{ay} = -\frac{2i}{\ln(L_c/a)}\int_0^\infty \mathrm{d}r\left[\frac{1}{r}\tilde{\Psi}'_{-1}(r) + 2\nu\tilde{\omega}_{-1}(r)\right] \ , \qquad \text{[S57]}$$

where Ψ is the stream function and $\tilde{}_n$ correspond to the nth angular Fourier moment. Using the relation $\nabla^2\Psi = -\omega$, along with the fact that $\Psi'_{-1}(0) = (i/2)(u_{0x} + iu_{0y})$ where $\mathbf{u}_0$ is the active flow evaluated at the defect core, we obtain $\int_0^\infty \mathrm{d}r\tilde{\omega}_{-1} = i(u_{0x} + iu_{0y})$. This yields

$$U_{ax} + iU_{ay} = \frac{1}{\ln(L_c/a)}(u_{0x} + iu_{0y})\left[\ln\left(\frac{R}{a}\right) + 4\nu\right] - \frac{2i}{\ln(L_c/a)}\left(\int_0^R \frac{\mathrm{d}r}{r}[\tilde{\Psi}'_{-1}(r) - \tilde{\Psi}'_{-1}(0)] + \int_R^\infty \frac{\mathrm{d}r}{r}\tilde{\Psi}'_{-1}(r)\right) \ , \qquad \text{[S58]}$$

where R is some intermediary scale (or the tweezer radius in the case of finite size active tweezer patterns) whose precise value is irrelevant in calculating the nonsingular part of the final integral. The active defect motion ($\mathbf{U}_a$) is proportional to the active flow at the core ($\mathbf{u}_0$) as long as $R \sim L_c \gg ae^{-4\nu}$ and $\tilde{\Psi}'_{-1}$ decays sufficiently rapidly for $r \gg R$, so the final integrals can be neglected. This is the expected behavior in the frictionally dominated regime where flow impacts defect motion locally. When viscous stresses dominate, the final integral contribution from the streamfunction can dominate and the flow has more long-ranged consequences on defect motion. A detailed exploration of the impact of viscous effects on defect motion in the presence of activity gradients (generalizing work in Ref. (S15)) is left for future work.

5. Active defect hydrodynamics

In this section, we include interactions between defects and coarse-grain their dynamics to obtain defect hydrodynamic equations to describe an interacting defect gas. We generalize previous results by some of us (S3, S16) by also incorporating the propulsive dynamics of both $\pm 1/2$ defects in slowly varying activity gradients.

For a dilute gas of $\pm 1/2$ defects that are in slowly varying spatial activity pattern, we can assume the dynamics of defects is slow relative to the nematic relaxation time ($\tau_n = \gamma/a_2$). Within a mean-field description, we can incorporate interactions by considering each defect as moving in an background nematic texture that is quasistatically determined by all other defects. With these assumptions, we can build a particle-like description for active defects (see Sec. 4 for details on assumptions). Here we simply write down a phenomenological model for the defect dynamics based on previous works (S3, S5, S15, S17).

Defects are assumed to be advected by their local flow velocity at the core ($\mathbf{u}_0$) along with passive elastic interactions mediated by nematic distortions. Writing the local phase gradient as $\mathbf{v}_n = \boldsymbol{\nabla}\theta$, we write the positional dynamics of $\pm 1/2$ defects as

$$\dot{\mathbf{r}}^{\pm} = \mathbf{u}_0^{\pm} \pm \mu K \boldsymbol{\epsilon} \cdot \mathbf{v}_n \; , \tag{S59}$$

where $\boldsymbol{\epsilon}$ is the Levi-Civita tensor, $\mu \propto 1/\gamma$ is the defect mobility, and K is the Frank elastic constant. We neglect retardation and memory effects, and evaluate the local flow velocity ($\mathbf{u}_0^{\pm}$) at the instantaneous defect position ($\mathbf{r}^{\pm}(t)$). More details on the approximations entering the effective particle-like description of defect dynamics is provided in Sec. 4.

Orientational dynamics is similarly obtained by assuming defects rotate due to local vorticial flow at the defect core (ω_0) along with active torques that align defect motion with its orientation (S3, S15). Upon including noisy reorientations to model a finite persistence of motile defects, we write the rotational dynamics of $\pm 1/2$ defects as

$$\dot{\theta}_0^{\pm} = -\mu_R \mathbf{u}_0^{\pm} \cdot \mathbf{v}_n + \frac{1}{2}\omega_0^{\pm} + \sqrt{\frac{1}{2\tau_R}}\,\eta(t) \; , \tag{S60}$$

where $\eta(t)$ is unit-white Gaussian noise, τ_R is the defect persistence time ($D_R = 1/\tau_R$ is the rotational diffusion constant of the defect), and μ_R is the defect rotational mobility (dimensionless).

To write the defect dynamics in terms of activity and its gradients, we work in the frictional limit and use the expressions derived in Sec. D for $\mathbf{u}_0^{\pm}$ and $\omega_0^{\pm}$. Retaining leading contributions to flow and vorticity from activity gradients, we obtain (for extensile activity, $\alpha < 0$)

$$\dot{\mathbf{r}}^{+} = \boldsymbol{\mathcal{V}}_{+} \cdot \hat{\mathbf{e}} + \mu K \boldsymbol{\epsilon} \cdot \mathbf{v}_n \; , \tag{S61}$$

$$\dot{\mathbf{r}}^{-} = \boldsymbol{\mathcal{V}}_{-} : \boldsymbol{\Theta} - \mu K \boldsymbol{\epsilon} \cdot \mathbf{v}_n \; , \tag{S62}$$

$$\boldsymbol{\mathcal{V}}_{+} = -\left(v - \frac{7\ell^2}{2}\nabla^2 v\right)\mathbf{1} - 5\ell^2 \boldsymbol{\nabla}\boldsymbol{\nabla} v \; , \tag{S63}$$

$$\boldsymbol{\mathcal{V}}_{-} = 4\ell^2 \boldsymbol{\nabla}\boldsymbol{\nabla} v \; . \tag{S64}$$

where $v(\mathbf{r}) = |\alpha(\mathbf{r})|/(2a\Gamma)$ sets the scale of defect self-propulsion speed and $\ell = \sqrt{3aR}/2$ is left as a phenomenological length scale that controls the scale over which the defect probes the activity gradient. We will simply fit for the value of ℓ in the hydrodynamic equations (see Sec. 6 for details). The orientational dynamics of the defects can be similarly obtained as

$$\dot{\psi}_{+} = -2\mu_R \mathbf{v}_n \cdot \boldsymbol{\mathcal{V}}_{+} \cdot \hat{\mathbf{e}} + \hat{\mathbf{z}} \cdot (\boldsymbol{\Omega}_{+} \times \hat{\mathbf{e}}) + \sqrt{\frac{2}{\tau_R}}\,\eta_{+}(t) \; , \tag{S65}$$

$$\dot{\psi}_{-} = -\frac{2\mu_R}{3}\mathbf{v}_n \cdot \boldsymbol{\mathcal{V}}_{-} : \boldsymbol{\Theta} - \frac{1}{3}\mathrm{Re}[i\Theta_3 \Omega_{-}] + \frac{1}{3}\sqrt{\frac{2}{\tau_R}}\,\eta_{-}(t) \; , \tag{S66}$$

$$\boldsymbol{\Omega}_{+} = -3\boldsymbol{\nabla} v \; , \tag{S67}$$

$$\Omega_{-} = -5a^2 \partial^3 v \; , \tag{S68}$$

where we have used the fact that $\psi_{+} = 2\theta_0$ for $+1/2$ defects, but $\psi_{-} = 2\theta_0/3$ for $-1/2$ defects. The noise terms $\eta_{\pm}$ are both unit-Gaussian white noise that are independent of each other.

Following the procedure described in Ref. (S16), we coarse-grain these dynamical equations for defects as active quasiparticles (Eqs. S61-S66) into hydrodynamic equations for an interacting defect gas. We define defect densities and currents as

$$\rho_{\pm}(\mathbf{r}, t) = \left\langle \sum_{\mu} \delta[\mathbf{r} - \mathbf{r}_{\mu}^{\pm}(t)] \right\rangle \; , \quad \mathbf{j}_{\pm}(\mathbf{r}, t) = \left\langle \sum_{\mu} \dot{\mathbf{r}}_{\mu}^{\pm}(t)\delta[\mathbf{r} - \mathbf{r}_{\mu}^{\pm}(t)] \right\rangle \; , \tag{S69}$$

where $\mathbf{r}_{\mu}^{\pm}(t)$ is the position of the μth $\pm 1/2$ defect. We also have the defect number ($n = (\rho_{+} + \rho_{-})/2$) and charge ($\rho = (\rho_{+} - \rho_{-})/2$) densities along with the defect number ($\mathbf{j}_n = (\mathbf{j}_{+} + \mathbf{j}_{-})/2$) and charge ($\mathbf{j}_{\rho} = (\mathbf{j}_{+} - \mathbf{j}_{-})/2$) currents (the factor

of $1/2$ in all the definitions comes because the defects have charge $|\nu| = 1/2$). The coarse-grained defect orientational order parameters (for both $\pm 1/2$ defects) are defined as

$$\mathbf{p}(\mathbf{r},t) = \left\langle \sum_\mu \hat{\mathbf{e}}_\mu(t)\delta[\mathbf{r} - \mathbf{r}_\mu^+(t)] \right\rangle \ , \quad T_{ijk}(\mathbf{r},t) = \left\langle \sum_\mu \Theta^\mu_{ijk}(t)\delta[\mathbf{r} - \mathbf{r}_\mu^-(t)] \right\rangle \ . \tag{S70}$$

For the $-1/2$ defects, we will also use the equivalent complex representation for the triatic order parameter $T_3(\mathbf{r},t) = \left\langle \sum_\mu \Theta_3^\mu(t)\delta[\mathbf{r} - \mathbf{r}_\mu^-(t)] \right\rangle$. The defect densities satisfy the following balance equations

$$\partial_t n + \boldsymbol{\nabla}\cdot\mathbf{j}_n = W_c - W_a \ , \quad \partial_t \rho + \boldsymbol{\nabla}\cdot\mathbf{j}_\rho = 0 \ , \tag{S71}$$

where $W_{c/a}$ are creation/annihilation rates and the coarse grained constitutive relations for the currents are given by

$$\mathbf{j}_\rho = \frac{1}{2}\boldsymbol{\mathcal{V}}_+\cdot\mathbf{p} - \frac{1}{2}\boldsymbol{\mathcal{V}}_- : \mathbf{T} + n\mu K\boldsymbol{\epsilon}\cdot\mathbf{v}_n - D_0\boldsymbol{\nabla}\rho \ , \tag{S72}$$

$$\mathbf{j}_n = \frac{1}{2}\boldsymbol{\mathcal{V}}_+\cdot\mathbf{p} + \frac{1}{2}\boldsymbol{\mathcal{V}}_- : \mathbf{T} + \rho\mu K\boldsymbol{\epsilon}\cdot\mathbf{v}_n - D_0\boldsymbol{\nabla} n \ , \tag{S73}$$

where we have included a finite translational diffusion constant (D_0) that we retain for numerical stability when we solve for the steady state profiles plotted in Fig. 3 (Main text). Note the mean defect density ($n \propto |\alpha| \sim v$) in steady state is set by the balance of defect creation-annihilation rates ($W_c = W_a$) which dictates the mean separation between defects $\xi_d \sim 1/\sqrt{n}$ (S18, S19). The phase gradient satisfies the topological conservation (Gauss) law as before: $\hat{\mathbf{z}}\cdot(\boldsymbol{\nabla}\times\mathbf{v}_n) = 2\pi\rho$ (S16, S20). The nonlinear hydrodynamic equations for the defect orientational order parameters ($\mathbf{p}$ and T_3) are given by

$$\partial_t\mathbf{p} + \frac{1}{2}\boldsymbol{\nabla}\cdot(\rho_+\boldsymbol{\mathcal{V}}_+) = -\frac{1}{\tau_R}\mathbf{p} - \frac{1}{2}\rho_+\boldsymbol{\Omega}_+ + \rho_+\mu_R\boldsymbol{\mathcal{V}}_+\cdot\boldsymbol{\epsilon}\cdot\mathbf{v}_n + D_0\nabla^2\mathbf{p} \ , \tag{S74}$$

$$\partial_t T_3 + \bar{\partial}[\rho_-\bar{\mathcal{V}}_-] = -\frac{1}{\tau_R}T_3 - \frac{1}{2}\rho_-\bar{\Omega}_- - i\rho_-\mu_R\bar{\mathcal{V}}_-(v_{nx} + iv_{ny}) + D_0\nabla^2 T_3 \ . \tag{S75}$$

where now in complex notation $\bar{\mathcal{V}}_- = 4\ell^2\bar{\partial}^2 v$. In deriving Eqs. S81, S82, we only assume there is no large scale defect ordering (i.e., the defect gas is globally isotropic) allowing a simple closure where we set $\langle\sum_\mu \hat{\mathbf{e}}_\mu\hat{\mathbf{e}}_\mu\delta[\mathbf{r} - \mathbf{r}_\mu^+]\rangle = (\rho_+/2)\mathbf{I}$ and $\langle\sum_\mu(\Theta_3^\mu)^2\delta[\mathbf{r} - \mathbf{r}_\mu^-]\rangle = 0$ (no nematic ordering of $+1/2$ defects and no hexatic ordering of $-1/2$ defects). We have additionally included a small diffusion constant (D_0) for numeircal stability and smoothness reasons.

Note, in Eqs. S81, S82, three separate effects allow for the collective reorientation of the defects in activity gradients - the first is simply through a differential translational flux akin to an 'active pressure' (second term on the LHS), the second is due to a finite active vorticity (second term on RHS) and finally an active self-induced torque that is a collective effect (present only when $\mathbf{v}_n \neq 0$). For $+1/2$ defects, the first two of these terms are of the same sign and cause $+1/2$ defects to align parallel to activity gradients. For $-1/2$ defects, the first two terms oppose each other, though the active vorticity term is a factor $a/\xi_d \ll 1$ smaller. For simplicity we shall neglect the active vorticity term $\Omega_- \ll \Omega_+$ in Eq. S82 for now.

Eqs. S71-S82 complete the active defect hydrodynamic model. In a 1D extensile activity pattern $v = v(x)$, at steady state ($\partial_t = 0$), we write $\mathbf{p} = p(x)\hat{\mathbf{x}}$, $\mathbf{v}_n = v_n(x)\hat{\mathbf{y}}$, etc. The steady-state defect number density (n) depends on the local activity as it is determined by the balance of the creation and annihilation rates ($W_c = W_a$). We check that contributions from gradients of the number current $\mathbf{j}_n$ are negligible and $n = n(x)$ is a fixed function of the local activity $v(x)$ (with $n(x) \propto v(x)$ for large $v(x)$, see Sec. 6 and Fig. S5A for details). Upon solving Eqs. S81, S82 and neglecting diffusion ($D_0 \sim 0$), we obtain,

$$\frac{1}{\tau_R}p(x) = -\frac{1}{2}\partial_x(\rho_+\mathcal{V}_+) - \frac{1}{2}\rho_+\Omega_+ + \mu_R\mathcal{V}_+\rho_+ \ v_n \ , \tag{S76}$$

$$\frac{1}{\tau_R}T_3(x) = -\frac{1}{2}\partial_x(\rho_-\mathcal{V}_-) + \mu_R\mathcal{V}_-\rho_- \ v_n \ , \tag{S77}$$

where the only nonvanishing components of the response coefficients $\boldsymbol{\mathcal{V}}_\pm, \boldsymbol{\Omega}_\pm$ are $\mathcal{V}_+(x) = -[v(x) + c_0\ell^2 v''(x)]$, $\mathcal{V}_-(x) = \ell^2 v''(x)$, $\Omega_+(x) = -c_+v'(x)$ (for extensile activity, $\alpha < 0$). While our simplified calculation yields values $c_0 = 3/2$, $c_+ = 3$ and $\mu_R = 1$, we leave these parameters as phenomenological coefficients and fit for them using simple activity patterns (see Sec. 6 for details). The charge density is given by $\rho(x) = \partial_x v_n/(2\pi)$ and the phase gradient is determined by the vanishing of the charge current $\mathbf{j}_\rho = \mathbf{0}$ (Eq. S83), which gives (again neglecting diffusion, $D_0 \sim 0$),

$$\mathcal{V}_+(x)p(x) - T_3(x)\mathcal{V}_-(x) + 2\mu K n(x) \ v_n(x) = 0 \ . \tag{S78}$$

In Sec. 6, we describe the procedure for solving these steady-state equations and fitting the unknown parameters to compare with the numerical simulations.

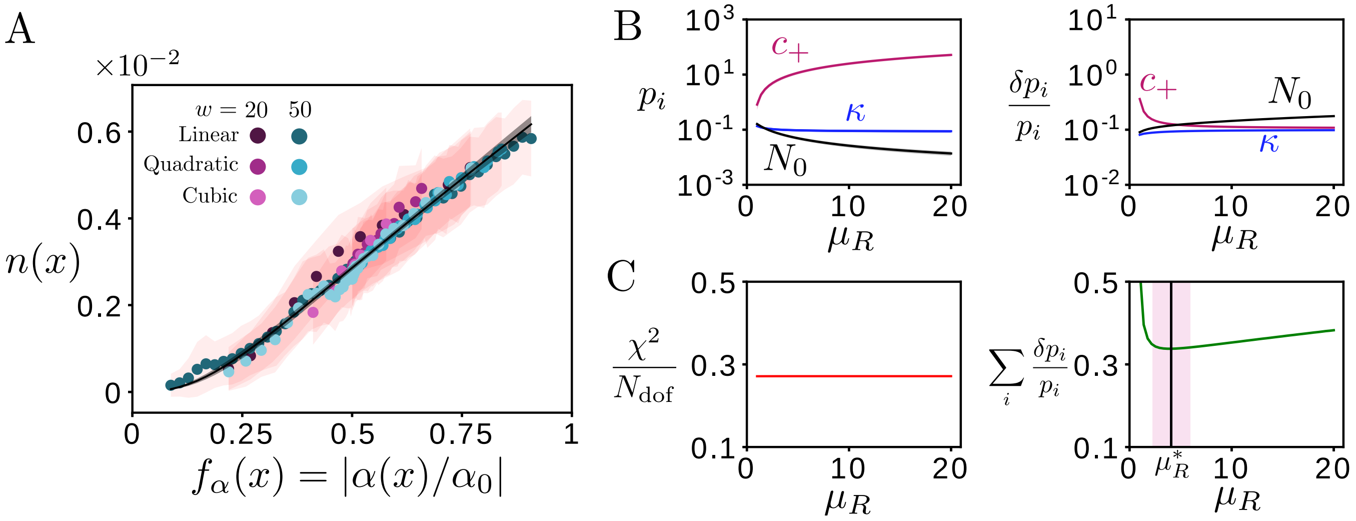

Fig. S5. (A) The defect number density $n(x)$ is determined by the local activity and is proportional to $|\alpha(x)|$ for large activity. The scatter plot includes data (in dots) for different polynomial activity profiles (linear, quadratic and cubic) with two different widths $w = 20, 50$. The overlapping shaded pink regions represents one standard deviation in the numerical measurements for different cases. The black line shows the optimal curve fitted to Eq. S86 with the (very small) shaded gray region showing one standard deviation of the fit (computed using a synthetic ensemble with $N = 500$ using the Gaussian distributed parameters centered on the optimal fit values and using the fit covariance matrix). (B) The best fit values for the parameters $p_i = \kappa, N_0, c_+$ (left) obtained using the linear activity profile data, as a function of μ_R which is varied. On the right are plotted the relative standard deviation ($\delta p_i/p_i$) of the fit parameters as a function of μ_R. (C) The $\chi^2/N_{\rm dof}$ for the fit to the linear activity profile data is largely independent of μ_R (left). On the right, the sum of the relative standard deviation ($\sum_i \delta p_i/p_i$) is plotted as a function of μ_R. This curve attains a minimum at $\mu_R = \mu_R^*$ (vertical black line) and the standard deviation (estimated using the jackknife method) is shown as the shaded region around μ_R^*.

6. Estimating defect hydrodynamic parameters

We fit and constrain the parameters in the defect hydrodynamic equations using steady-state distributions of the defect density n, the charge density ρ, and the $\pm 1/2$ orientational order parameters $(\mathbf{p}, T_3)$. The nonlinear defect hydrodynamic equations in the presence of activity gradients described above are reproduced below for convenience,

$$\partial_t n + \boldsymbol{\nabla}\cdot\mathbf{j}_n = W_c - W_a\ , \qquad \text{[S79]}$$

$$\partial_t \rho + \boldsymbol{\nabla}\cdot\mathbf{j}_\rho = 0\ , \qquad \text{[S80]}$$

$$\partial_t \mathbf{p} + \frac{1}{2}\boldsymbol{\nabla}\cdot(\rho_+\boldsymbol{\mathcal{V}}_+) = -\frac{1}{\tau_R}\mathbf{p} - \frac{1}{2}\rho_+\boldsymbol{\Omega}_+ + \rho_+\mu_R\boldsymbol{\mathcal{V}}_+\cdot\boldsymbol{\epsilon}\cdot\mathbf{v}_n + D_0\nabla^2\mathbf{p}\ , \qquad \text{[S81]}$$

$$\partial_t T_3 + \bar{\partial}[\rho_-\bar{\mathcal{V}}_-] = -\frac{1}{\tau_R}T_3 - \frac{1}{2}\rho_-\bar{\Omega}_- - i\rho_-\mu_R\bar{\mathcal{V}}_-(v_{nx} + iv_{ny}) + D_0\nabla^2 T_3\ . \qquad \text{[S82]}$$

where the phase gradient $\mathbf{v}_n$ is related to the charge density by topological (Gauss) conservation law: $\hat{\mathbf{z}}\cdot(\boldsymbol{\nabla}\times\mathbf{v}_n) = 2\pi\rho$, and the density and charge currents ($\mathbf{j}_n, \mathbf{j}_\rho$ respectively) are given by

$$\mathbf{j}_\rho = \frac{1}{2}\boldsymbol{\mathcal{V}}_+\cdot\mathbf{p} - \frac{1}{2}\boldsymbol{\mathcal{V}}_- : \mathbf{T} + n\mu K\boldsymbol{\epsilon}\cdot\mathbf{v}_n - D_0\boldsymbol{\nabla}\rho\ , \qquad \text{[S83]}$$

$$\mathbf{j}_n = \frac{1}{2}\boldsymbol{\mathcal{V}}_+\cdot\mathbf{p} + \frac{1}{2}\boldsymbol{\mathcal{V}}_- : \mathbf{T} + \rho\mu K\boldsymbol{\epsilon}\cdot\mathbf{v}_n - D_0\boldsymbol{\nabla}n\ . \qquad \text{[S84]}$$

In order to estimate the various parameters in Eqs. S79-S84, we use simple 1D polynomial activity profiles to fit the various fields. The activity is given by

$$\frac{\alpha(x)}{\alpha_0} = H(x) + \frac{1}{2}[1 - H(x)]\left[1 + \left(\frac{x}{w}\right)^m\right]\ , \qquad \text{[S85]}$$

where $m = 1, 2, 3$, $H(x) = (1/2)[1 + \tanh(|x| - w)]$, the activity scale $\alpha_0 = -5$ (in units where nematic relaxation time and coherence length are both unity) and $w = 20, 50$ controls the length scale of the gradient. We will only consider the spatial interval $x \in [-(w - \delta w), w - \delta w]$ with $\delta w = 8$ where effects from the sigmoidal profile at the interface can be neglected and the activity profile is just a simple polynomial.

We assume the defect density (n) rapidly relaxes to its stationary value given by the balance of creation and annihilation rates ($W_c = W_a$, neglecting $\boldsymbol{\nabla}\cdot\mathbf{j}_n$ gradient terms) which only depend on the local activity. Plotting the averaged total defect number density $n(x)$ as a function of the local nondimensionalized activity $f_\alpha(x) = |\alpha(x)/\alpha_0| = (1 + x/w)^m$ for the various polynomial profiles ($m = 1, 2, 3$), we obtain data collapse (Fig. S5A) which can be fit to a simple curve

$$n(x) = n_0\frac{f_\alpha^3(x)}{b + f_\alpha(x)^2}\ , \qquad \text{[S86]}$$

 Suraj Shankar, Luca V. D. Scharrer, Mark J. Bowick, M. Cristina Marchetti

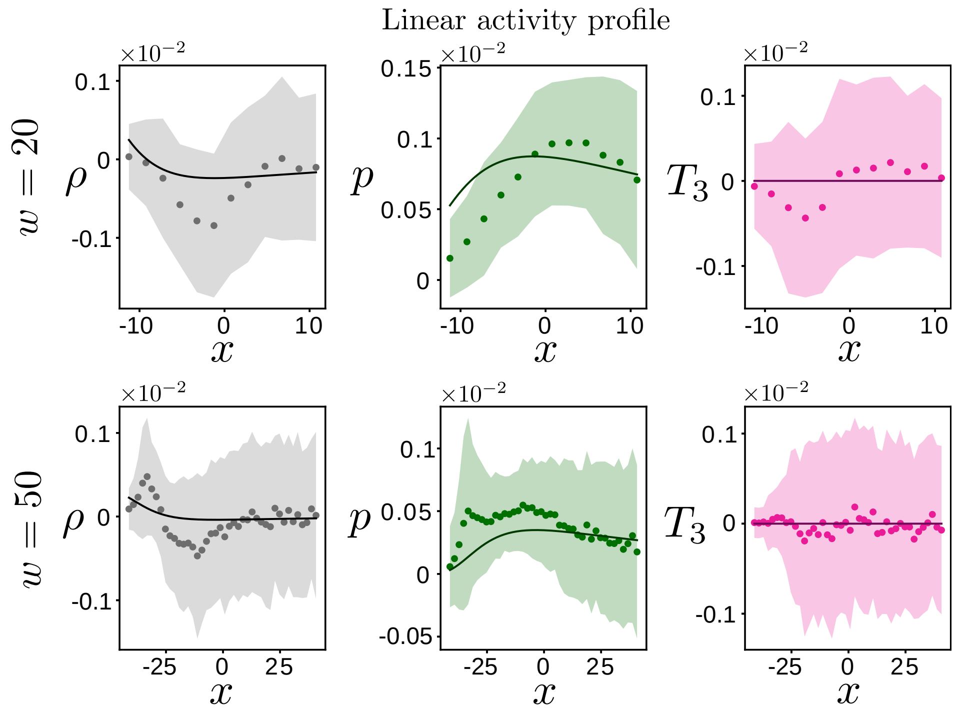

Fig. S6. Fitted spatial profiles (solid curves) and numerically computed profiles (dots: average, shaded region: standard deviation) of fields $\rho(x)$ (left), $p(x)$ (middle) and $T_3(x)$ (right), for a linear activity profile with width $w = 20$ (top) and $w = 50$ (bottom).

with parameters $n_0 = (7 \pm 0.4) \times 10^{-3}$ (maximal defect density) and $b = (7 \pm 2) \times 10^{-2}$, as shown in Fig. S5A. The nonlinear fit is weighted using the standard deviations in the numerically computed density, using SciPy's curve_fit function. The functional form in Eq. S86 is chosen so that at high activity we recover the known linear scaling of defect density with activity ($n(x) \sim n_0 f_\alpha(x)$) (S18, S19) and for low activity, the density vanishes as a power law in the activity. We can perform a three parameter fit as well, including the exponent so that $n = n_0 (f_\alpha)^{\beta+1}/[b + (f_\alpha)^\beta]$, and we obtain large errors in b and $\beta = 1.9 \pm 0.5$, consistent with the chosen functional form. By fixing $\beta = 2$, we obtain consistent parameter values but with smaller errors. The standard deviation for the fitted curve is estimated using a synthetic ensemble of $N = 500$ realizations of curves with random parameters normally distributed with mean given by the optimal fit values and standard deviation given by the fit covariance matrix. The resulting sample standard deviation is shown as a gray shaded region in Fig. S5A.

Using the fitted form of $n(x)$ from Eq. S86, we solve the Eqs. S80-S82 at steady state ($\partial_t = 0$). As the imposed activity gradients are weak ($|p|, |T_3|, |\rho| \ll n$), we linearize the equations, neglect $\Omega_- \ll \Omega_+$ and neglect diffusive terms by setting $D_0 = 0$, which yields the following linearized steady-state equations

$$-\frac{1}{\tau_R} p - \frac{1}{2}\partial_x (n\mathcal{V}_+) - \frac{1}{2} n\Omega_+ + \mu_R \mathcal{V}_+ n \, v_n = 0 \, , \qquad \text{[S87]}$$

$$-\frac{1}{\tau_R} T_3 - \frac{1}{2}\partial_x (n\mathcal{V}_-) + \mu_R \mathcal{V}_- n \, v_n = 0 \, , \qquad \text{[S88]}$$

$$p\mathcal{V}_+ - T_3 \mathcal{V}_- + 2\mu K n \, v_n = 0 \, , \qquad \text{[S89]}$$

with $\mathcal{V}_+(x) = -v_0[f_\alpha(x) + c_0 \ell^2 f''_\alpha(x)]$, $\mathcal{V}_-(x) = v_0 \ell^2 f''_\alpha(x)$, $\Omega_+(x) = -c_+ v_0 f'_\alpha(x)$ (written for extensile activity, $\alpha < 0$), where $v_0 > 0$ sets the scale of the self-propulsion speed, ℓ is a length scale over which defects probe activity gradients and c_0, c_+ are phenomenological constants. The charge density is computed using $\rho(x) = \partial_x v_n/(2\pi)$. As Eqs. S87-S89 are algebraic and linear, we can directly solve for $p(x)$, $T_3(x)$ and $\rho(x)$ in a given polynomial activity profile. The steady-state solutions depend on the following 6 parameters

$$\kappa = \frac{2\mu K}{\tau_R \mu_R v_0^2} \, , \quad N_0 = n_0 v_0 \tau_R \, , \quad c_+ \, , \quad c_0 \, , \quad \mu_R \quad \text{and} \quad \ell \, , \qquad \text{[S90]}$$

where κ, c_0, c_+, μ_R are dimensionless, ℓ is a length and N_0 is a linear density (dimensions of inverse length). The latter two parameters are reported in units of the nematic coherence length $\xi = 1$.

We perform sequential multiparamter fits of the numerically computed $\rho(x)$, $p(x)$, $T_3(x)$ fields (in the spatial interval $x \in [-(w - \delta w), w - \delta w]$, $\delta w = 8$ to avoid interface effects) to the linearized analytical solutions obtained by solving Eqs. S87-S89. All fits are performed using a custom Python code for constrained, (variance) weighted least-squares minimization (method='trf') using SciPy's curve_fit function. The numerically obtained spatial profiles of $\rho(x)$, $p(x)$ and $T_3(x)$ along with the fitted curves are plotted in Fig. S6 (linear activity profile) and Fig. S7 (quadratic activity profile). For a linear activity profile

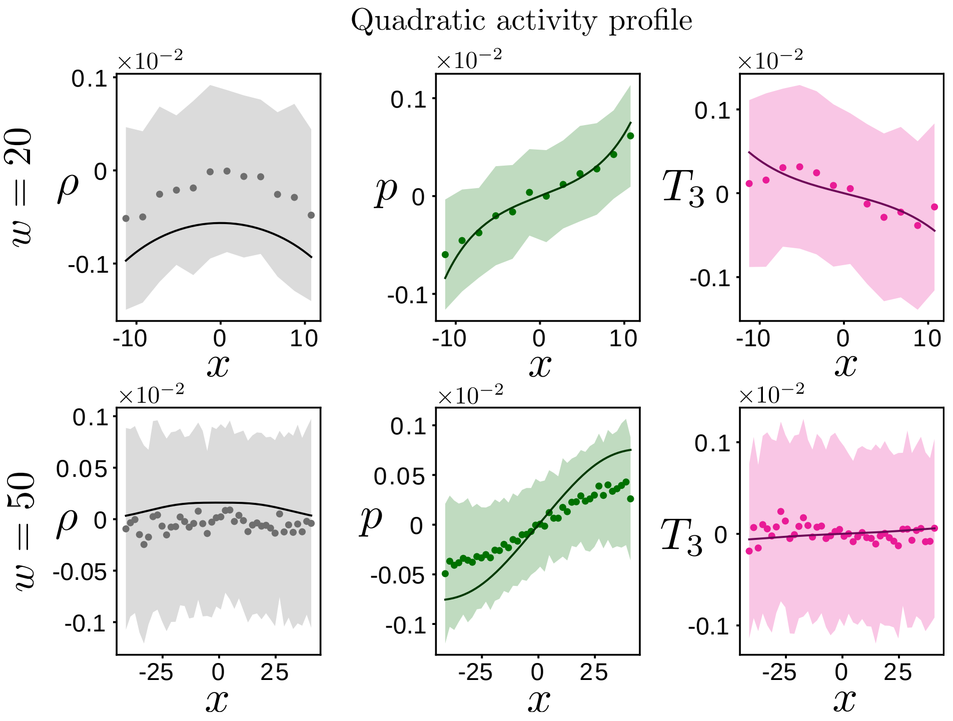

Fig. S7. Fitted spatial profiles (solid curves) and numerically computed profiles (dots: average, shaded region: standard deviation) of fields $\rho(x)$ (left), $p(x)$ (middle) and $T_3(x)$ (right), for a quadratic activity profile with width $w = 20$ (top) and $w = 50$ (bottom).

($f_\alpha(x) = 1 + x/w$) $\mathcal{V}_- = 0$, $\mathcal{V}_+ = -v_0(1 + x/w)$ and $\Omega_+ = -c_+ v_0/w$, so $T_3 = 0$ (Eq. S88) and the solutions are independent of c_0 and ℓ. The model is still sloppy (S21) when fitting for the remaining 4 parameters: κ, N_0, c_+ and μ_R. In particular μ_R is found to be the least constrained parameter. To resolve this issue, we perform simultaneous least-square minimization for two linear activity profiles ($w = 20, 50$) with 3 fitting parameters (κ, N_0, c_+) and fixed μ_R that we later vary in the range $\mu_R \in [1, 20]$ (see Fig. S5B, left). To account for the variability in the density curves ($n(x)$), for each value of μ_R, we additionally generate a synthetic ensemble of $n(x)$ profiles ($N_b = 50$) with Gaussian distributed b (mean: 7×10^{-2}, standard deviation: 2×10^{-2}, ensuring only $b > 0$ is chosen), and perform the same fit with the independent $n(x)$ functions as input. The reported standard deviation (plotted relative to the optimal parameter value, $\delta p_i/p_i$, in Fig. S5B, right) on each fitted parameter ($p_i = \kappa, N_0, c_+$) is computed as the square root of the sum (over the $N_b = 50$ ensemble) of the fit variance - this assumes the errors are independent Gaussian random variables. The fit quality is nearly independent of μ_R as quantified by χ^2/N_{dof} (Fig. S5C, left), reflecting the sloppy nature of the model. So we instead look at the total relative error ($\sum_i(\delta p_i/p_i)$ - sum of standard deviation relative to parameter value, summed over the fitted parameters) and find the optimal $\mu_R = \mu_R^*$ at which $\sum_i(\delta p_i/p_i)$ is minimized (Fig. S5C, right). The standard deviation in the optimal value of $\mu_R = \mu_R^*$ (shown as shaded region in Fig. S5C, right) is estimated using the jackknife resampling approach over the $N_b = 50$ data set.

To now obtain the values of ℓ and c_+, we perform a constrained least-square fitting simultaneously for two different quadratic activity profiles ($f_\alpha(x) = (1 + x/w)^2$ with $w = 20, 50$. For each sample $n(x)$ from the $N_b = 50$ ensemble, we use a jackknife block average for μ_R (i.e., $\mu_R = \mu_{R,i}^{jk}$ where $\mu_{R,i}^{jk} = \sum_{j=1,j\neq i}^{N_b} \mu_{R,j}^*/(N_b - 1)$ and $\mu_{R,j}^*$ is the optimal value minimizing the relative error for that realization of $n(x)$), recompute the fit parameters κ, N_0, c_+ for the linear gradient and use these parameter values in the least-squares minimization for the quadratic profile. This then gives a distribution of fit parameters ℓ, c_+ for each value in the $N_b = 50$ ensemble. The ensemble distribution of all fit parameters is shown in Fig. S8.

To produce the plots in Fig. 3 of the main text, we use the active strip profile (Eq. S10) and solve the nonlinear dynamical equations in Eqs. S80-S82 along with the Gauss constraint and use the above obtained fit parameters. The resulting curves are averaged over the parameter distribution across the $N_b = 50$ ensemble. Note that directly solving the linearized steady-state equations (Eqs. S87-S89) with the activity profile in Eq. S10 for interface widths $w = 15, 40$ leads to singular and unphysical solutions as sharp interfacial gradients can cause $|\rho(x)| > n(x)$ implying an unphysical negative density of $\pm 1/2$ defects, i.e., $\rho_\pm(x) < 0$. To avoid this issue, we instead solve the nonlinear dynamical equations (Eqs. S79-S82) to steady-state.

We discretize and numerically solve the defect hydrodynamic equations using the finite element method in FEniCS (S22, S23) with spatial nodes $N_{\mathrm{nodes}} = 32L$ ($2L = 128$ is the box size), second-order Lagrange (P2) elements, and time step $\mathrm{d}t = 5 \times 10^{-3}\tau_R$ (results largely unchanged for $\mathrm{d}t = 10^{-2}\tau_R - 10^{-3}\tau_R$) for a total run time of $T = 10^3\tau_R$. We integrate the equations in time using a second order semi-implicit Crank-Nicholson and Adams-Bashforth scheme with Neumann boundary conditions and enforce the $\rho_\pm(x)$ positivity constraint by setting $j_\rho(x) = 0$ when $|\rho(x)| > n(x)$. We additionally include the diffusive D_0 term to stabilize and smooth the numerical integration with an estimated diffusion constant $D_0 = \ell_p^2/(2\tau_R)$ where the defect persistence length $\ell_p = v_0\tau_R = N_0/n_0 \approx 7$ ($N_0 \simeq 5 \times 10^{-2}$, $n_0 \simeq 7 \times 10^{-3}$). The numerically computed curves are averaged

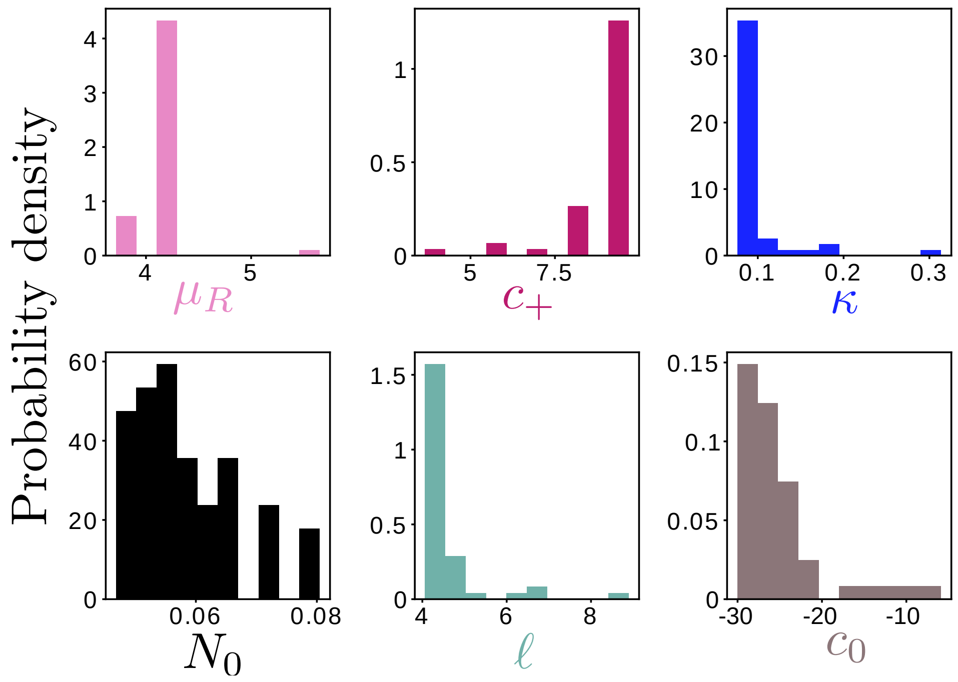

Fig. S8. The distribution of obtained fit parameters over the $N_b = 50$ ensemble.

over the $N_b = 50$ parameter ensemble and plotted on the interval $x \in [-64, 64]$ with a $N_{\text{plot}} = 1200$ equispaced points.

Using the numerical solution of the hydrodynamic equation we compute the interfacial dipole moment $D = (1/2)\int \mathrm{d}x\ |x|\rho(x)$ for interfaces of different widths ($w = 5-70$). To estimate the standard deviation of the dipole moment, we use the bootstrap method and randomly choose an $N = 10$ subsample of the fitted parameter set (from the $N_b = 50$ ensemble) and compute the average and standard deviation for the subsample.

The only parameter that we cannot obtain from fitting steady-state distributions is the defect reorientation time τ_R. Note the numerical integration of the defect hydrodynamic equations are performed in time steps that are in units of τ_R, so the steady-state properties are independent of the time scale. To obtain an estimate of τ_R, we measure the defect orientational correlation function from the full numerical nematodynamic simulations. For simplicity we focus on $+1/2$ defects in a nematic with uniform activity ($\alpha_0 = -5$, same as the maximal activity in all the collective patterning results). We initialize the simulation with a random nematic texture, and let it evolve for 30 time units, saving the positions and orientations of $+1/2$ defects at each timestep. For each individual $+1/2$ defect present during this timespan, we calculate the orientational time autocorrelation function $\mathcal{C}(t) = \langle \hat{\mathbf{e}}_i(T_i^c) \cdot \hat{\mathbf{e}}_i(T_i^c + t) \rangle$, with $0 < \delta t < T_i^a - T_i^c$, where T_i^c and T_i^a are the creation and annihilation times (so $T_i^a - T_i^c$ is the survival lifetime) of the ith defect respectively. The correlation function is averaged over all defects and time (green dots in Fig. S9, shaded region is standard deviation) and fit to an exponentially decaying function (e^{-t/τ_R}, black line in Fig. S9) to extract an estimate for $\tau_R \approx 11$. In the analysis of the dynamic response of defects to oscillating interfacial gradients (Fig. 4, main text), we can then estimate the average time $\tau = \bar{w}^2/D_a$ it takes $+1/2$ defects to cross the interface, where the active diffusion constant $D_a = \langle |\mathbf{u}_0|^2 \rangle \tau_R/2$. At the interface, we assume a linear gradient of active velocity $|\mathbf{u}_0| \sim v_0 \delta x/w$ ($\delta x \in [0, w]$ within the interface), which upon averaging over the interface width gives $\langle |\mathbf{u}_0|^2 \rangle \sim v_0^2/3$. Using $\tau_R = 10$ and persistence length $\ell_p = v_0\tau_R = 7$, we then obtain $D_a = v_0^2\tau_R/6 \approx 0.82$, which gives a characteristic traversal time $\tau \sim 765$ (with average width ($\bar{w} = 25$)).

7. Supplementary Movies

Movie S1. Demonstration of an active topological tweezer transporting a $-1/2$ defect (magenta triangle) along a bent trajectory (Fig. 2A-C). Both normalized vorticity (ω, top left) and flow speed (u, bottom left) are plotted. The activity pattern actuated in the tweezer is shown on the top-right and the defect trajectory is plotted on the bottom-right. The tweezer protocol details are given in Table S1.

Movie S2. Demonstration an active topological tweezer transporting a $+1/2$ defect (green arrow) along a bent trajectory (Fig. 2D-F). Both normalized vorticity (ω, top left) and flow speed (u, bottom left) are plotted. The activity pattern actuated in the tweezer is shown on the top-right and the defect trajectory is plotted on the bottom-right. The tweezer protocol details are given in Table S2.

Movie S3. Demonstration of controlled defect pair nucleation, braiding and pair exchange using active topological tweezers (Fig. 2G-I). The normalized vorticity (ω), flow speed (u) and defect space-time trajectories

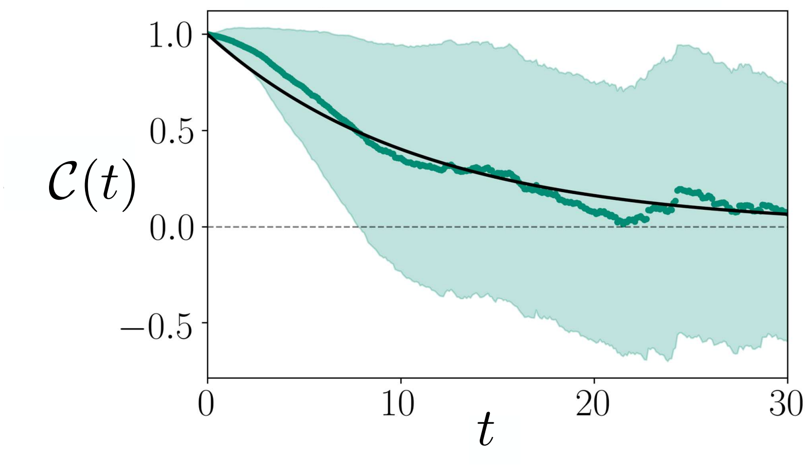

Fig. S9. $+1/2$ defect orientational time correlation function $\mathcal{C}(t)$ plotted against time (t) for spatially homogeneous activity $\alpha = -5$. The numerically computed correlation function is plotted in green with the shaded region showing the standard deviation. In black is a fit to an exponential function (e^{-t/τ_R}) which gives the $+1/2$ defect reorientation time as $\tau_R \approx 11$.

are plotted. The tweezer protocol details are given in Tables S3-S5.

Movie S4. Collective patterning of defects using an active strip (Fig. 3C) showing both the normalized vorticity (ω) and flow speed (u), along with the static activity profile ($\alpha(x)$). Shallow interfacial gradients ($w = 40$, $\alpha_0 = -5$) allow $+1/2$ defects to escape through and get trapped on the passive sides. This results in a charge polarized interface with a positive dipole moment.

Movie S5. Collective patterning of defects using an active strip (Fig. 3D) showing both the normalized vorticity (ω) and flow speed (u), along with the static activity profile ($\alpha(x)$). Sharp interfacial gradients ($w = 15$, $\alpha_0 = -5$) entrap $-1/2$ defects at the interface preventing the escape of motile $+1/2$ defects. This results in an inverted polarized interface with a negative dipole moment.

Movie S6. Slow dynamic response of defects in an active strip with oscillating gradients (Fig. 4). Both normalized vorticity (ω) and flow speed (u) are shown along with the dynamic activity profile ($\alpha(x,t)$). Slow sinusoidal oscillations of the interface width ($f\tau \sim 0.7$) allow the defects to reorganize quasi-adiabatically and provide enough time for the $+1/2$ defects to escape outside the strip. This results in a conventionally polarized interface with a time-averaged positive dipole moment.

Movie S7. Rapid dynamic response of defects in an active strip with oscillating gradients (Fig. 4). Both normalized vorticity (ω) and flow speed (u) are shown along with the dynamic activity profile ($\alpha(x,t)$). Fast sinusoidal oscillations of the interface width ($f\tau \sim 1.3$) do not provide enough time for the $+1/2$ defects to escape outside the strip. This results in the defects getting dynamically trapped within the strip leading to an inverted polarized interface with a time-averaged negative dipole moment.

Movie S8. Collective transport of active defects (Fig. 5) with a slowly moving active strip ($V = 0.025$). Both normalized vorticity (ω) and flow speed (u) are shown along with the dynamic activity profile ($\alpha(x,t)$). Slow motion of the strip allows the defect distribution to equilibribiate within the strip and get transported quasi-adiabatically. As a result, negligible number of defects leak and escape out of the strip, but the transport task takes a very long time (asymptotically infinite as $V \to 0$).

Movie S9. Optimal collective transport of active defects (Fig. 5) with an active strip moving at intermediate speeds ($V = 1$). Both normalized vorticity (ω) and flow speed (u) are shown along with the dynamic activity profile ($\alpha(x,t)$). At the optimal speed, the motion of the active strip matches the self-propulsion speed of the $+1/2$ defects allowing the defects to collectively 'surf' the travelling activity pattern. This allows the transport task to be completed in a finite time, with minimal leakage and escape of defects from the active strip.

Movie S10. Failed collective transport of active defects (Fig. 5) with a fast moving active strip ($V = 2$). Both normalized vorticity (ω) and flow speed (u) are shown along with the dynamic activity profile ($\alpha(x,t)$). At very large speeds, the activity patterns moves too quickly to provide suffiecient time to even nucleate enough

defect pairs and transport them. Any transiently created defect pairs rapidly leave the active region and passively annihilate before they can be transported.

References

[S1] AJ Vromans, L Giomi, Orientational properties of nematic disclinations. *Soft matter* **12**, 6490–6495 (2016).
[S2] X Tang, JV Selinger, Orientation of topological defects in 2d nematic liquid crystals. *Soft matter* **13**, 5481–5490 (2017).
[S3] S Shankar, S Ramaswamy, MC Marchetti, MJ Bowick, Defect unbinding in active nematics. *Phys. review letters* **121**, 108002 (2018).
[S4] S Shankar, A Souslov, MJ Bowick, MC Marchetti, V Vitelli, Topological active matter. *Nat. Rev. Phys.* **4**, 380–398 (2022).
[S5] L Giomi, MJ Bowick, X Ma, MC Marchetti, Defect annihilation and proliferation in active nematics. *Phys. review letters* **110**, 228101 (2013).
[S6] L Giomi, MJ Bowick, P Mishra, R Sknepnek, M Cristina Marchetti, Defect dynamics in active nematics. *Philos. Transactions Royal Soc. A: Math. Phys. Eng. Sci.* **372**, 20130365 (2014).
[S7] L Pismen, Dynamics of defects in an active nematic layer. *Phys. Rev. E* **88**, 050502 (2013).
[S8] J Rønning, MC Marchetti, L Angheluta, Defect self-propulsion in active nematic films with spatially-varying activity (2022).
[S9] P Srivastava, P Mishra, MC Marchetti, Negative stiffness and modulated states in active nematics. *Soft matter* **12**, 8214–8225 (2016).
[S10] SP Thampi, R Golestanian, JM Yeomans, Active nematic materials with substrate friction. *Phys. Rev. E* **90**, 062307 (2014).
[S11] R Voituriez, JF Joanny, J Prost, Spontaneous flow transition in active polar gels. *Europhys. Lett.* **70**, 404 (2005).
[S12] L Giomi, L Mahadevan, B Chakraborty, M Hagan, Banding, excitability and chaos in active nematic suspensions. *Nonlinearity* **25**, 2245 (2012).
[S13] CC Tsai, Solutions of slow brinkman flows using the method of fundamental solutions. *Int. journal for numerical methods fluids* **56**, 927–940 (2008).
[S14] J Rønning, CM Marchetti, MJ Bowick, L Angheluta, Flow around topological defects in active nematic films. *Proc. Royal Soc. A* **478**, 20210879 (2022).
[S15] L Angheluta, Z Chen, MC Marchetti, MJ Bowick, The role of fluid flow in the dynamics of active nematic defects. *New J. Phys.* **23**, 033009 (2021).
[S16] S Shankar, MC Marchetti, Hydrodynamics of active defects: From order to chaos to defect ordering. *Phys. Rev. X* **9**, 041047 (2019).
[S17] X Tang, JV Selinger, Alignment of a topological defect by an activity gradient. *Phys. Rev. E* **103**, 022703 (2021).
[S18] L Giomi, Geometry and topology of turbulence in active nematics. *Phys. Rev. X* **5**, 031003 (2015).
[S19] EJ Hemingway, P Mishra, MC Marchetti, SM Fielding, Correlation lengths in hydrodynamic models of active nematics. *Soft Matter* **12**, 7943–7952 (2016).
[S20] V Ambegaokar, B Halperin, DR Nelson, ED Siggia, Dynamics of superfluid films. *Phys. Rev. B* **21**, 1806 (1980).
[S21] RN Gutenkunst, et al., Universally sloppy parameter sensitivities in systems biology models. *PLoS computational biology* **3**, e189 (2007).
[S22] A Logg, KA Mardal, G Wells, *Automated solution of differential equations by the finite element method: The FEniCS book.* (Springer Science & Business Media) Vol. 84, (2012).
[S23] M Alnæs, et al., The fenics project version 1.5. *Arch. Numer. Softw.* **3** (2015).
